# Genetic, parental and lifestyle factors influence telomere length

**DOI:** 10.1038/s42003-022-03521-7

**Published:** 2022-06-09

**Authors:** Sergio Andreu-Sánchez, Geraldine Aubert, Aida Ripoll-Cladellas, Sandra Henkelman, Daria V. Zhernakova, Trishla Sinha, Alexander Kurilshikov, Maria Carmen Cenit, Marc Jan Bonder, Lude Franke, Cisca Wijmenga, Jingyuan Fu, Monique G. P. van der Wijst, Marta Melé, Peter Lansdorp, Alexandra Zhernakova

**Affiliations:** 1 Department of Genetics, University of Groningen, University Medical Center Groningen, Groningen, the Netherlands; 2 Department of Pediatrics, University of Groningen, University Medical Center Groningen, Groningen, the Netherlands; 3 Terry Fox Laboratory, British Columbia Cancer Research Center, Vancouver, BC Canada; 4 Repeat Diagnostics Inc, Vancouver, BC Canada; 5 Life Sciences Department, Barcelona Supercomputing Center, 08034 Barcelona, Catalonia Spain; 6 European Research Institute for the Biology of Ageing, University of Groningen, Groningen, the Netherlands; 7 Laboratory of Genomic Diversity, Center for Computer Technologies, ITMO University, St. Petersburg, 197101 Russia; 8 Microbial Ecology, Nutrition, and Health Research Unit, Institute of Agrochemistry and Food Technology (IATA-CSIC), 46980 Paterna-Valencia, Spain; 9 Division of Computational Genomics and Systems Genetics, German Cancer Research Center (DKFZ), 69120 Heidelberg, Germany; 10 European Molecular Biology Laboratory, Genome Biology Unit, 69117 Heidelberg, Germany; 11 Departments of Hematology and Medical Genetics, University of British Columbia, Vancouver, BC Canada

**Keywords:** Senescence, Lymphocytes

## Abstract

The average length of telomere repeats (TL) declines with age and is considered to be a marker of biological ageing. Here, we measured TL in six blood cell types from 1046 individuals using the clinically validated Flow-FISH method. We identified remarkable cell-type-specific variations in TL. Host genetics, environmental, parental and intrinsic factors such as sex, parental age, and smoking are associated to variations in TL. By analysing the genome-wide methylation patterns, we identified that the association of maternal, but not paternal, age to TL is mediated by epigenetics. Single-cell RNA-sequencing data for 62 participants revealed differential gene expression in T-cells. Genes negatively associated with TL were enriched for pathways related to translation and nonsense-mediated decay. Altogether, this study addresses cell-type-specific differences in telomere biology and its relation to cell-type-specific gene expression and highlights how perinatal factors play a role in determining TL, on top of genetics and lifestyle.

## Abstract (web summary)

Deep molecular and phenotypic data highlights the links of human telomere lengths from six different blood cells with genetics, parental phenotypes mediated by epigenetic signals and expression changes at the single cell level.

## Introduction

With an increasingly ageing worldwide population, age-related diseases pose a great burden in clinical care and socioeconomics. Healthy ageing is the goal to counter this trend, but this term is complex and not easily defined. Several markers for premature or delayed ageing have been suggested, including telomere length^1,2^, DNA methylation^3,4^ and thymic function^5^. Telomeres are repetitive DNA structures located at the chromosome ends and, together with their associated proteins, play a fundamental role in chromosomal stability^6,7^. Telomeres are known to decrease with age^2,8^ as a result of multiple factors, including limiting telomerase activity^9^, the end-replication problem^1^, end-processing^10^, and oxidative stress^11^. Both genetic and environmental factors are known to influence telomere length. Several genetic loci have been associated with telomere length^12–16^, with heritability estimates ranging from 34%^17^ to 82%^18,19^. However, heritability estimates often cannot distinguish between true genetic determinants and early life factors such as parental or environmental exposures that could affect telomere length during adulthood^18^. This is especially true in twin-based studies, where the early life exposures are confounded with genetic effects.

Importantly, most telomere length analyses carried out to date focused on blood leukocytes and did not explore cell-type- and tissue-specific variability. A recent study examining the variability of telomere length in a wide range of post-mortem tissues^20^ showed that, although whole blood telomere measurements might be a proxy for and synchronous with those of other tissues^21^, there are significant tissue-specific differences. However, this post-mortem tissue study did not address the possibility of telomere length differences between different cell types within a tissue. In addition, most large studies to date have used PCR or Luminex-based methods to measure relative telomere length in isolated genomic DNA, and these approaches have shown reproducibility biases^22,23^ that could potentially explain the heterogeneous and contradicting findings^24^.

Telomere length may have important physiological consequences^25^. It has been proposed that telomere length might regulate gene expression^26–29^ but also that gene expression can directly contribute to telomere length attrition or conservation^30,31^. Given the observed variability in telomere length with cell population^20^, it is conceivable that this will be related with cell-type-specific expression patterns, which have not been investigated to date.

Here, we explore telomere variation using Flow-FISH telomere length measurements in Lifelines Deep (LLD), a well-characterised population cohort from the Netherlands^32^. We measured telomere length in six different cell types in 1046 participants. By combining this data with genetic information available for LLD participants and with rich phenotypic information that includes blood cell counts and immune markers, self-reported diseases, birth-related phenotypes, parental diseases and behaviour, epigenomics profiles and single-cell expression patterns in 62 individuals, we determined the major contributors to telomere length variation. Specifically, we studied: (1) the difference in telomere length across the six blood cell types, (2) the relationship between leukocyte telomere length with other ageing markers, (3) the correlation of telomere ageing markers with biochemical, parental and clinical phenotypes and mortality, (4) the contribution of genetic and non-genetic factors to variations in telomere length and (5) the cell-type-specific changes in gene expression associated with cell-type-specific telomere length variation, which may pinpoint major functional pathways related to telomere variability.

## Results

### Telomere length captures biological variability other than age

**Fig. 1:**
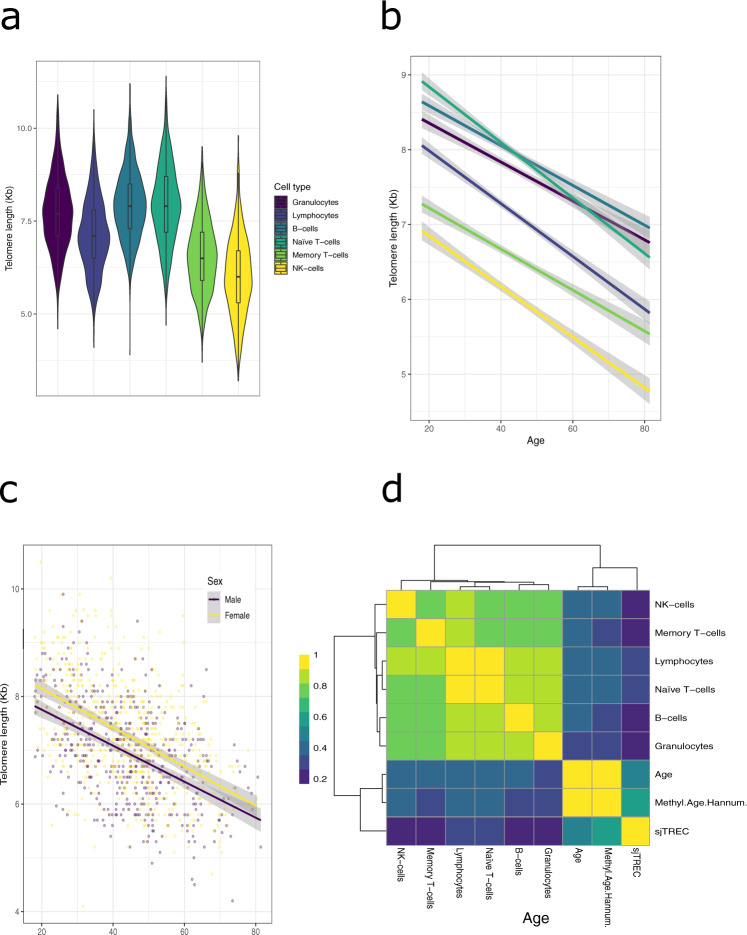
Telomere measurements in six blood cell types. **a** Distribution of telomere length by cell type. **b** Average trend line of telomere length. Grey shading indicates 95% confidence interval. Length decreases with age in all six cell types. Colour indicates cell type in both (**a**, **b**). **c** Sexual dimorphism of telomere length (shown in lymphocytes). Trend line indicates average length per age. Grey shading indicates 95% confidence interval. **d** Heatmap displaying the absolute correlation value (Spearman’s rho) between telomere lengths, chronological age (Age), Hannum-based methylation age and sjTRECs' qPCR relative expression.

LLD^32^ is a population cohort from the northern Netherlands that includes participants with a wide age range (mean 43.9 years ± 13.7 sd, min 18.0, max 81.4) for whom we have deep phenotypic and molecular information [available data illustrated in Supplementary Fig. 1, for descriptive statistics see Supplementary Data 1]. In 1046 LLD participants, Flow-FISH^33^ was used to measure the telomere length of six blood cell types: granulocytes, lymphocytes, B-cells (CD45RA+CD20+ ), naïve T-cells (CD45RA+CD20−), memory T-cells (CD45RA−) and NK-cells/fully differentiated T-cells (CD45RA+CD57+) (hereafter referred to as NK-cells) [Fig. 1a]. We found that all six cell-type-specific telomere lengths decreased with age [Fig. 1b] and were, on average, shorter in males than females over the entire age range [Fig. 1c] [Supplementary Fig. 2b for all cell types]. We also observed a similar moderate negative correlation between age and telomere length among cell types (Pearson correlation, maximum *r* = −0.43, minimum *r* = −0.33) [Fig. 1d]. These findings agree with those of a Flow-FISH‒based study in a North American cohort^9^, although the two cohorts differed in their participant recruitment selection criteria. Nevertheless, in the overlapping age ranges, both studies find comparable telomere lengths [Supplementary Fig. 2a], which supports the accuracy of our measurements. We observe that naïve T-cells and B-cells have the longest telomeres on average, whereas NK- and memory T-cells show significantly shorter telomeres than other cell types (paired Wilcoxon-test, *p* < 2 × 10^−16^). While naïve T-cells showed the highest mean telomere length [Fig. 1a], they also showed the largest negative association of telomere length and age (linear model, slope of −0.034), from an average 8.59 Kb in the <32.9 years age group (first quartile) to an average 7.32 Kb in individuals >52.7 years (fourth quartile) [Fig. 1b]. The rate of telomere loss we observe in naïve T-cells matched previous observations^9^ and does not support production of naïve T-cells from more primitive precursors after puberty and thymus involution^34^. Instead, we assume that naïve T-cells are maintained after puberty by homeostatic mechanisms that are likely to involve cell divisions that result in telomere loss, but these processes are currently poorly understood.

Next, we compared telomere length with other biological age markers, including the methylation-based Hannum^35^ age-index and signal joint T-cell receptor excision circles (sjTRECs) expression (CT values of a qPCR), which represents thymic TCR maturation for a given individual^36^. Here, we observed that both methylation age and sjTREC were more strongly associated with chronological age than telomere length, and neither was highly correlated with telomere length [Fig. 1d]. After removing the variability attributed to chronological age, methylation age and sjTREC were negatively associated (Pearson correlation, *r* = −0.36), but we found no association to telomere length. Telomere lengths remained highly correlated between different cell types [Supplementary Fig. 2d].

Overall, these findings suggest that telomere length captures biological variation other than chronological age, and the source of this variation is distinct compared to other ageing markers measured, specifically methylation age and thymic function.

### Genetic contribution to telomere length

**Fig. 2:**
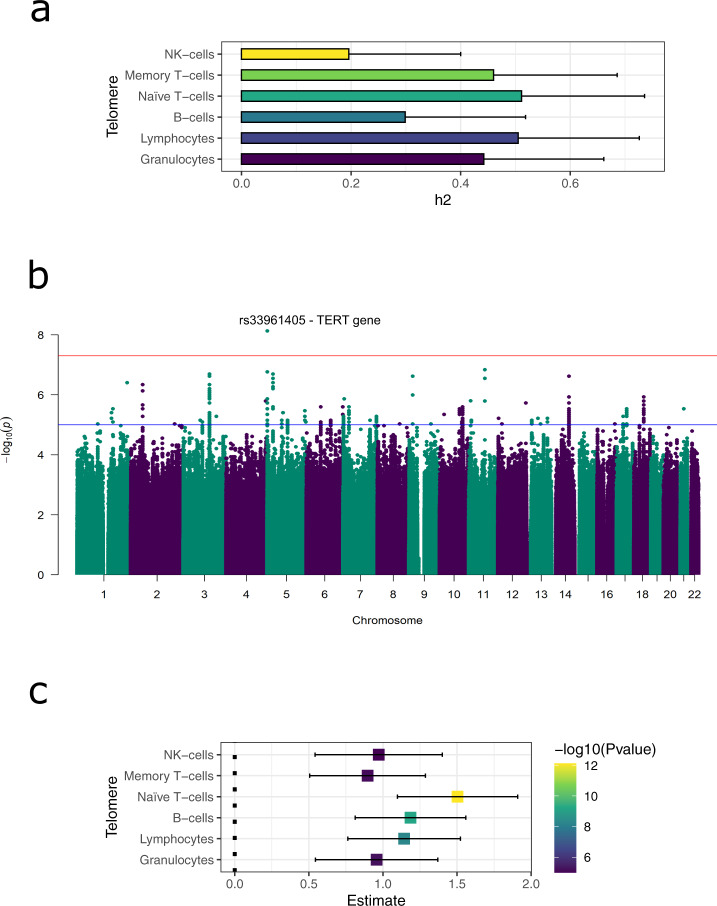
Genetic determinants of telomere length. **a** Heritability estimation using a GREML model on the genotyped SNP data, from which a kinship matrix was estimated using GCTA^40^. The kinship matrix was used in a mixed model to estimate the variation explained by the genetic random effect. Units of heritability are represented as h2. **b** Manhattan plot of genome-wide associations to telomere length from all cell types. Blue line indicates a suggestive significance threshold of *p* = 1 × 10^−5^. Red line indicates the standard genome-wide threshold of *p* = 5 × 10^−8^. The novel association with *TERT*, rs33961405 (5:1277577:G:A GRCh37), is labelled with its corresponding rsid. **c** Correlation of the Polygenic Risk Score (PRS) of telomere length^15^ and telomere length of different cell types. Each square represents the estimated effect of the PRS in the cell type. Bars indicate 95% confidence interval.

To explore to what extent genetics can explain variation in telomere length, we first performed a heritability analysis. We used genotype data to infer genetic relations and fit a GREML model, while controlling for age and sex. This analysis provides an estimate of the total telomere length variability attributable to the additive effects of common genetic variants. The results show a median SNP-based heritability of 45%, with a maximum of 51% (naïve T-cells) and a minimum of 19.6% (NK-cells). Although the standard errors of the estimates were high, with a median of 22% [Fig. 2a], the heritability estimate falls in a similar range to those of previous reports^37–39^ and the corresponding *P* values were below 0.05 for all cell types except NK-cells (*p* = 0.15). This result supports the idea that genetic factors contribute significantly to telomere length and can partially explain the inter-individual variability. The higher genetic contribution to naïve T-cell telomere length that we observe might be explained by the fact that these cells reflect less environmental influence. On the other hand, environmental factors will impact telomeres in memory T-cells, including NK-cells, because antigen-mediated clonal expansion of these cells is typically triggered by environmental factors (e.g., infections), and thus telomere length will decrease due to the number of replications.

To further explore the genetic contribution to telomere length, we performed a genome-wide association study (GWAS) of ~7.5 million genotyped or imputed SNPs on telomere length in each cell type. First, we tested if we could replicate previously published loci associations. For this, we used the 20 genome-wide significant loci from a recent meta-analysis published by ref. ^15^. Of the 17 published SNPs for which we also had information, six were replicated in at least one cell type with *p* < 0.05 and consistent allelic direction [Supplementary Data 2]. Similarly, we could replicate 6/12 European-associated genome-wide significant loci from a large trans-ethnic study^16^ [Supplementary Data 2]. We then looked at *de novo* associations and identified one significant association after applying permutation-based false discovery rate (FDR) correction: The A allele of rs33961405 (1000 Genomes European population allele frequency = 0.49) located in an intron of the telomerase reverse transcriptase (*TERT*) gene was associated with decreased telomere length of T-cells (effect size = −0.19, SE = 0.03) [Fig. 2b] [Supplementary Fig. 3] (summary statistics of associations *p* < 1 × 10^−5^ presented in [Supplementary Data 3]). Other genetic variants located near the *TERT* gene were previously found to affect leukocyte telomere length; however, rs33961405 is novel and is in moderate linkage disequilibrium (LD) with a previously published lead SNP in *TERT* (rs2736100, LD r^2^ = 0.47 in 1000 Genomes, European population)^12,13^.

To reproduce previous GWAS associations  at cell type level, we computed a Polygenic Risk Score (PRS) from a large GWAS on telomere lengths^15^ (see Methods) and correlated it to our telomere length data. This analysis identified highly significant positive associations. The strongest genetic associations were seen in cell types with longer telomeres: naïve T-cells (linear model (lm), effect estimate = 1.5, *p* = 7.61 × 10^−13^) and B-cells (lm, effect estimate = 1.19, *p* = 6.78 × 10^−10^). Cell types with shorter telomeres, memory T-cells (lm, effect estimate = 0.89, *p* = 7.47 × 10^−6^) and NK-cells (lm, effect estimate = 0.97, *p* = 1 × 10^−5^), showed weaker associations of the genetic determinants. An exception to this trend was granulocytes, which showed a similar association range to B-cells and NK-cells (lm, effect estimate = 0.95, 6.02 × 10^−6^), despite having one of the longest average telomere lengths.

### Telomere ancestry—parental age and smoking contribute to an individual’s telomere length

We exploited the extensive phenotypic information available for LLD study participants to uncover which environmental factors are correlated with telomere length. The phenotypic information consisted of 90 different parameters, including blood parameters (e.g., leukocyte counts), anthropometric measurements (e.g., BMI), physiological parameters (e.g., blood pressure), various pre-existing diseases (e.g., hypertension or cancer) and lifestyle factors (e.g., smoking), as well as parental phenotypes and habits including parental diseases, smoking and age at participant’s birth [Supplementary Data 4].

To associate telomere length with different phenotypes, we built a linear model using telomere length as the dependent variable and the standardised phenotype measurement as the regressor, while controlling for age and sex. Of the non-genetic factors, blood cell counts were strongly associated with telomere lengths (Supplementary Data 5). Since cell counts might act as a confounder for other associations (as cell types have different telomere length and thus might confound the observed inter-individual differences), we included cell counts as covariates in the model. Using this new model, we identified 37 associations of 12 phenotypes with telomeres of any cell type using an FDR < 0.05 threshold (summary statistics can be found in Supplementary Data 6).

Several parental factors were consistently associated with telomere lengths in different cells. Smoking phenotypes such as ‘any parent smoking’, ‘father smoking during your childhood’ and ‘mother smoking during pregnancy’ were negatively associated with the telomere lengths of almost all cell types (with the exception of NK-cells). ‘Age of father when you were born’ and ‘age of mother when you were born’ were positively associated with the telomere lengths of four cell types. In a model combining both paternal and maternal age (on an l1 penalisation, see Methods), we found paternal age to shrink to near 0 while maternal age was kept constant (with the exception of granulocytes), highlighting that the effect of maternal age on telomere length is independent of paternal age effects. Significant negative correlations with smoking were only observed with the paternal, parental and maternal-during-pregnancy smoking habits. However, all cell types also showed nominally significant associations (*p* < 0.05) with maternal smoking. Maternal smoking associations showed weaker effect sizes than paternal (lm, 0.06 average difference, SE: 0.01, *p* = 3.2 × 10^−4^) and fewer participants had mothers who smoked than fathers who smoked (674 fathers vs. 381 mothers), factors which together may explain why maternal smoking did not reach FDR significance. Paternal and maternal smoking were, however, shown to have additive effects: A model that included a numeric variable describing the number of parents who were smokers showed stronger associations than binary smoking phenotypes (father, mother, or any parent).

We further analysed the associations to other available smoking phenotypes. Here, we found a negative effect (*p* < 0.05) of ‘father smoking’, ‘mother smoking’ and ‘mother smoking during pregnancy’ in all the cell types tested. In addition, passive smoking during an individual’s lifetime also influenced telomere length, with the factor ‘do people smoke near you at work’ associated (*p* < 0.05) with shorter telomeres in 5/6 cell types. Conversely, current smoking of the participant was not significantly associated with any cell type (*p* > 0.05) [Supplementary Fig. 4a].

In addition to parental associations, we found four negative associations with participant’s BMI and three with participant’s waist circumference. These negative associations with BMI and waist circumference could be driven by their correlations to other phenotypes. We, therefore, explored the association of BMI with other phenotypes in a larger cohort of 10,000 participants from the same population^41^ and observed a positive correlation between BMI and parental smoking (Pearson, *p* < 4.78 × 10^−6^) and a negative correlation between BMI and parental age (Pearson, *p* < 9.15 × 10^−7^). After accounting for potential confounding effects of parental age and smoking habits, the associations between telomere length and BMI phenotypes remained significant [Supplementary Fig. 4b]. This observation supports the conclusion that the associations of telomere length to BMI are not driven by the confounding effects of parental age or smoking.

In addition, we found one cell-specific (having one cell type below FDR < 0.05) positive association with poorly healing wounds (granulocytes) and three cell-specific negative associations with blood alpha-1 antitrypsin (AAT) (Memory T-cell), pulse rate (granulocytes) and weight (B-cells) [Fig. 2a] (summary statistics in Supplementary Data 6).

**Fig. 3:**
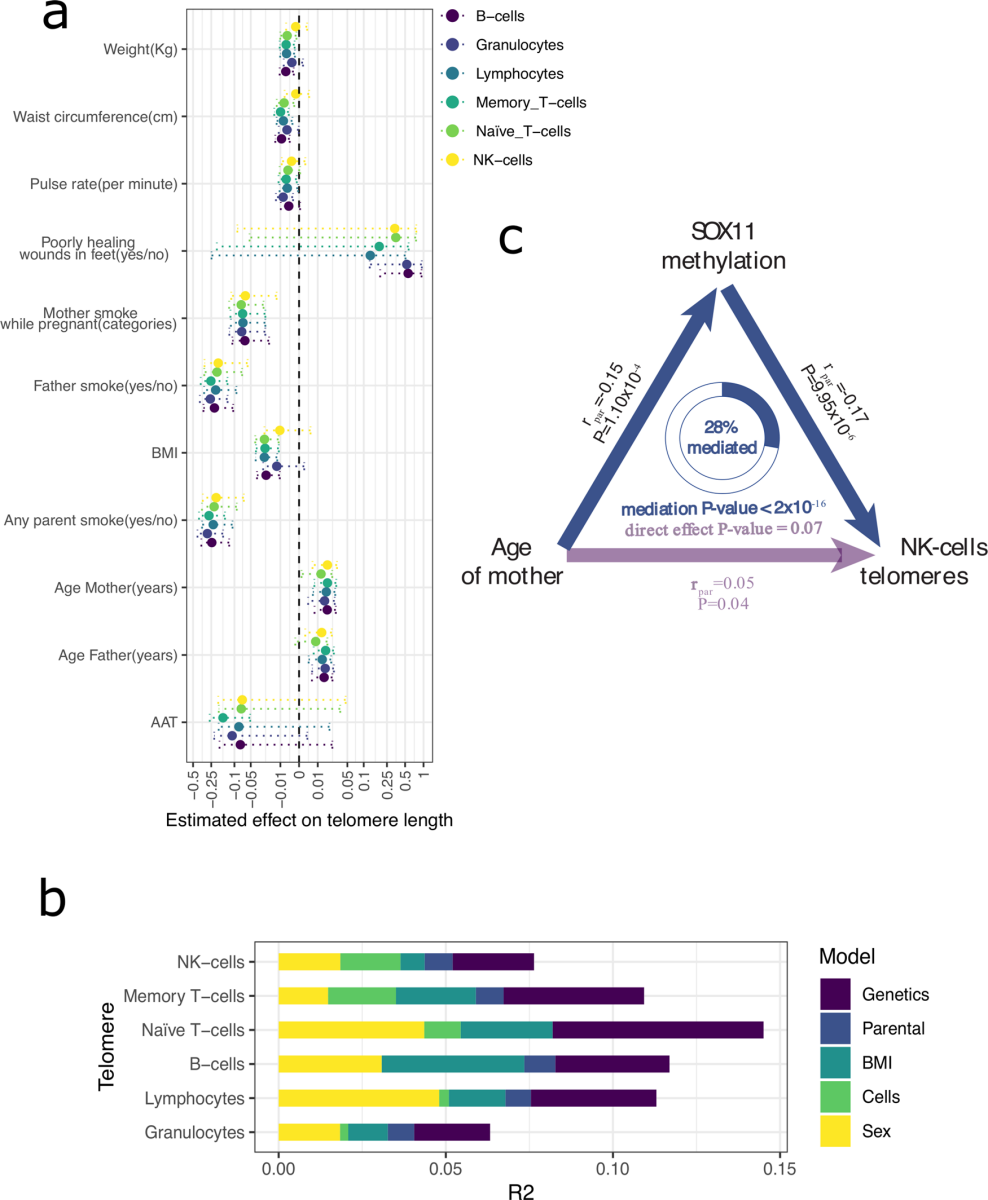
Non-genetic factors contribute to telomere variation. **a** Phenotype effect on telomere lengths of different cell types (with at least one significant association). Dashed lines show 95% confidence interval (estimate ± 2xSE). X-scale is symmetrical log-transformed (denominator constant = −2). BMI Body mass index, AAT Alpha-1 Antitrypsin Test. **b** Total variance of the telomere length explained after removing the effect of age. Only phenotypes associated with at least two cell types (FDR < 0.05) are used. Colour indicates the different partitions of variability. **c** Mediation effect of methylation of *SOX11* in the maternal age effect on NK-cell telomere length variability.

Finally, we assessed how much of the variation in telomere length not attributable to participant age could be explained by intrinsic, parental and genetic factors. For environmental factors, we included all associated factors with an FDR < 0.05 in at least two cell types. Genetic factors were addressed using PRS from a recently published telomere length GWAS^15^, see Methods. We fitted four nested models and estimated the added variability (R^2^) explained by each (see Methods) [Fig. 3b]. This revealed that most of the variability is explained by the addition of sex, BMI, waist circumference and cell counts (from 3.2 to 8%), depending on the cell type. The addition of parental phenotypes (parental age and smoking) added less information (0.8%), on average, than the multiple intrinsic factors. Finally, the contribution of genetics (average 3.7%) was lower than that of intrinsic factors (average 5.9%, considering sex as an intrinsic factor rather than a genetic one) in most cells, which is in line with the larger impact of environmental effects that we observed in the heritability analysis.

### Epigenetic changes may mediate the effect of parental phenotypes on telomere length

It has been proposed that maternal and paternal phenotypes, such as smoking habits and age at pregnancy, may affect a child’s phenotypes by inducing changes in methylation levels^42–47^. Several studies have reported an effect of parental age and smoking on the ‘methylation age’ of the child^43,44,46,48^. We, therefore, investigated whether the effect of parental phenotypes on telomere length could be mediated by changes in methylation levels. We first performed a GWAS of parental smoking and age with DNA methylation levels available for the same samples (*N* = 749). This resulted in 19 genome-wide significant associations of methylation probes with parental phenotypes at a Bonferroni-corrected *p* < 0.05 (summary statistics in Supplementary Data 7). Combining these results with the associations of parental phenotypes with telomere length (*p* < 0.05) and of telomere length with methylation (*p* < 0.05), we performed mediation analysis for the 17 resulting triplet associations. This analysis identified four triplets that showed significant mediation of the parental phenotype effect on telomere length through methylation of various probes (see Methods) [Supplementary Data 7]. All these significant mediation results were for the age of mother, even though we tested fewer triplets for maternal (6 triplets) than paternal age (11 triplets). Maternal age effects thus appear more likely to be mediated by methylation than paternal age effects.

One interesting example of this methylation mediation is for the SRY-related HMG-box gene *SOX11*. This gene is a known transcription factor and proliferation gene that plays an important role in embryonic development, cell fate determination and cancer. Our results indicate that the positive association of maternal age at birth with telomere length in NK-cells may be mediated by decreased methylation of CpG islands located in the promoter of *SOX11* (mediation *p* < 2 × 10^−16^), with up to 28% of the effect mediated through methylation of *SOX11* [Fig. 3c] [Supplementary Data 7].

### Telomere length changes show cell-type-specific associations with gene expression level

To study the relationships between telomere length variation with gene expression changes, we used single-cell RNA-sequencing (scRNA-seq) data generated on cryopreserved peripheral blood mononuclear cells (PBMCs) from 62 LLD donors, for which telomere length on six cell types was measured^49^. To classify cells, we used the high-resolution cell-type-annotations predicted by Azimuth^50^ to closely reflect the resolution of the Flow-FISH annotations (i.e., naïve and memory CD4T and CD8T cells, NK- and B-cells) (see Methods) (Supplementary Data 8). First, we confirmed that the subset of 62 LLD donors had similar telomere length distributions to the entire study population (1046 LLD donors) (Supplementary Fig. 5). We then performed telomere length differential gene expression analyses at single-cell resolution (sc-DEA) by selecting the matched telomere length measurement and gene expression level for each of these cell types (see Methods) (Supplementary Data 8). These analyses revealed *DNAJA1* to be positively associated with telomere length in memory CD8T cells (effect size = 0.05 log-fold change (LFC) per telomere length unit, FDR = 0.03) (Supplementary Data 9). *DNAJA1* encodes a heat shock protein 70 co-chaperone that was previously reported to bind telomeres in a study that used in vivo cross-linking, tandem affinity purification and label-free quantitative mass spectrometry^51^.

**Fig. 4:**
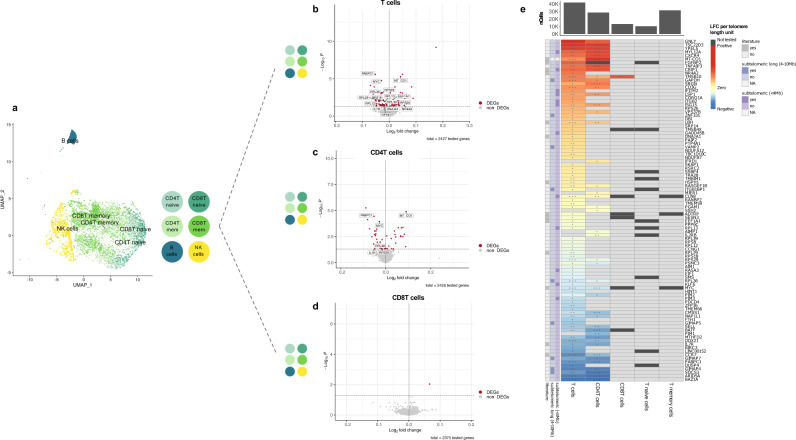
Differential gene expression changes with telomere length across cell types. **a** UMAP plot of the cells from the subset of 62 LLD donors with both scRNA-seq and Flow-FISH telomere length data. The cells are coloured by the cell type classification that closely reflects the resolution of the Flow-FISH annotations (i.e., naïve and memory -mem- CD4T and CD8T cells, NK- and B-cells). For visualisation purposes, we down-sampled each of Azimuth’s predicted cell types to 500 cells. **b**–**d** Volcano plots showing the results of DE approach II in T-, CD4T and CD8T cells, respectively. For DE approach II, we combined multiple cell types together (in **a**) in the same analysis (i.e., combining all T, all CD4T, all CD8T, all naïve T and all memory T-cells). DEGs with telomere length are represented in red. Non-DEGs are represented in grey. Labels correspond to the DEGs mentioned in the text. **e** Heatmap of log-fold change (LFC) per telomere length unit for the set of 97 unique DEGs identified in T-, CD4T and CD8T cells. Non-DEGs are shown in light grey. Genes not tested (i.e., those below 10% expression cut-off) are shown in dark grey. The significance level of the DEGs corresponds to the following arbitrary FDR thresholds: FDR < 1 × 10^−3^ (***), FDR < 0.01 (**) and FDR < 0.05 (*). The genes are sorted by their average LFC across cell types. The row annotation bars show whether the genes are located at the subtelomeric (<4 Mb) or subtelomeric long (4–10 Mb) region and if the genes were previously reported in any of the following studies: Pellegrino-Coppola et al.^53^, Tacutu R et al.^54^, Buxton JL et al.^29^ or Nittis T et al.^51^ (Supplementary Data 11). The distance to the telomeres was not calculated for the mitochondrial gene (MT-CO1) (subtelomeric (<4 Mb) and subtelomeric long (4–10 Mb) = NA). The column annotation on the bar plot shows the total number of cells (nCells) per cell type.

Because we only identified one differentially expressed (DE) gene, we wondered whether this could be due to insufficient statistical power. To address this, we performed differential gene expression analysis on T-cells combining multiple cell types together (Methods) while controlling for cell type annotation (Supplementary Data 10). This strategy increased the number of cells per donor and thus, statistical power. However, in contrast to analysing each cell type separately, genes identified with this combined strategy will likely have a similar association with telomere length across cell types. We identified 97 unique DE genes, one in CD8T cells, 44 in CD4T cells and 91 in all T-cells (Fig. 4) (Supplementary Data 11, Supplementary Fig. 6), including the *DNAJA1* association reported in our previous analysis.

Next, we explored potential mechanisms explaining the telomere length associations with expression in the set of 97 genes. Several mechanisms have been described by which telomere length may affect gene expression levels. The first is the telomere position effect (TPE)^26^. This mechanism results in decreased repression of genes located in the subtelomeric region when telomeres shorten^27^ and in subsequent overexpression, which is a process associated with CpG-methylation^29^. To determine whether our sets of DE genes were influenced by TPE, we tested whether there was an enrichment of DE genes in the subtelomeric region (< 4 Mb from chromosome end) (Supplementary Data 11). In CD4T cells, we found a significant enrichment of genes positively (5/19, *p* = 7 × 10^−3^) but not negatively associated (0/24, *p* = 1) with telomere length (Supplementary Fig. 7). We did not observe any enrichment in all T-cells (8/45, *p* = 0.05 for positively associated and 4/45, *p* = 0.58 for negatively associated genes) (Supplementary Data 11). The enrichment we observe in the CD4T cells is opposite to what we expected, i.e., we expected to identify genes that were negatively associated with telomere length as a result of the lost repression due to shortening. Our contrasting findings suggest that other mechanisms might be at work in the associated genes.

A second mechanism by which telomere length may affect gene expression levels occurs through a telomere position effect over long distances (TPE-OLD). Such long-distance gene expression regulation was shown to be the result of telomeres forming chromatin loops with enhancer and repressor regions of genes within 10 Mb of chromosome ends^28^. These interactions are dependent on telomere length but are not linearly related with shortening of the telomeres. We did not find any enrichment within our DE gene sets for genes acting through a potential TPE-OLD mechanism (i.e., 4–10 Mb from chromosome end) in either T-cells (4/45 DE genes positively associated with telomere length, *p* = 1; 5/45, DE genes negatively associated with telomere length, *p* = 0.8) or CD4 cells (1/19 DE genes positively associated with telomere length; *p* = 0.7; 4/24 DE genes negatively associated with telomere length, *p* = 0.52) (Supplementary Fig. 7) (Supplementary Data 11). However, the generalised linear mixed model (GLMM) we used for sc-DEA assumes linearity and therefore might not allow us to properly test this hypothesis, which assumes non-linear relations.

Since many of the identified DE genes did not fall into the TPE or TPE-OLD categories, other unknown effects on gene expression through telomere length may be at play or, conversely, the expression of specific genes may affect telomere length. For several DE genes, we found additional evidence for a telomere connection in literature (Fig. 4) (Supplementary Data 11). Firstly, three of our hits (*DNAJA1*, *EEF1A1* and *RPL29*) were previously reported in a screen for novel telomere binding proteins, indicating their direct involvement in telomere maintenance^51^. Secondly, CpG methylation of *NR4A2* was previously found to be associated with telomere length in whole blood^29^. *NR4A2* is involved in T-cell maintenance through regulation of Treg suppressor functions and repression of aberrant Th1 induction^52^. The third line of gene–telomere interaction evidence is the overlap with leukocyte ageing–associated genes that were previously identified in whole blood after a deep correction for cell type composition (8 out of 84 testable genes overlapped; odds ratio = 3.3, *p* = 6 × 10^−3^)^53^. Finally, four genes also overlapped with the GenAge human database, the core Human Ageing Genomic Resources (HAGR) database composed of >300 human ageing-related genes^54^. These four were related to immunosenescence^55,56^ (*IL7R*), cellular senescence^57,58^ (*MYC*) and longevity^59–62^ (*EEF1A1* and *MT-CO1*), in line with telomere shortening being a well-known hallmark of both cellular senescence and organismal ageing^63,64^. However, we did not find a significant overlap between the genes we identified and the ones reported in that study (odds ratio = 1.4, *p* = 0.53).

After exploring individual gene associations, we wondered if the identified DE genes belonged to similar functional pathways and could thus highlight the biological interplay between telomeres and gene expression. To explore this, we performed a functional enrichment analysis within the different sets of DE genes. In CD4T cells, the JAK-STAT signalling pathway was negatively associated with telomere length (enrichment ratio = 18.63, FDR = 4 × 10^−3^) (Supplementary Data 12). This pathway has previously been associated with telomerase regulation in haematologic malignancies^65^ and immunosenescence^56^. Among the DE genes involved in the JAK-STAT signalling pathway, interleukin-7 receptor (IL7R) plays a critical role in lymphoid cell development^66,67^, and its gene expression network has been proposed as a potential biomarker for healthy ageing^55^. In all T-cells, we identified 26 pathways enriched among the genes negatively associated with telomere length, many of them related to translation, including peptide chain elongation, eukaryotic translation elongation or termination and initiation, among others (Supplementary Data 12). Previous genetic screens in yeast^68–71^ and *Arabidopsis thaliana*^72^ identified ribosome biogenesis as one of the largest gene categories linked to telomere length. On top of that, in human fibroblasts, cellular senescence triggered through telomere shortening can diminish ribosome biogenesis, resulting in rRNA precursors and accumulation of ribosomal proteins (such as *RPL29*, which we found to decrease expression with telomere length in T-cells)^73,74^. In addition, our set of negatively enriched pathways in T-cells also revealed the nonsense-mediated decay (NMD) pathway (including *PAMPC1* expression^75^) that has been recently proposed to regulate the levels of specific mRNAs that are important for telomere functions^76^.

### Short telomere length associates with all-cause mortality, independent of age

**Fig. 5:**
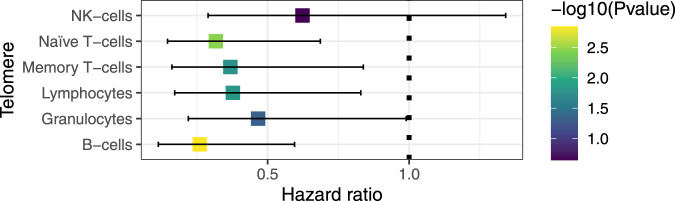
All-cause mortality risk of telomere length. *X* axis represents the hazard ratio of all-cause mortality estimated by a Cox regression on each cell type’s telomere length. Bars indicate estimated 95% confidence interval.

The most apparent implication of having abnormal telomere length may be its effect on morbidity and mortality. We collected all-cause mortality data for the previous 8 years, i.e., since the start of LLD data collection. Eleven study participants had died at the time of the analysis, and we used a Cox model while controlling for cell counts, age and sex to assess the predictive power of telomere length (using the survival time measured as days as an outcome). Despite the very low sample size, we found a consistent association of shorter telomeres and higher all-mortality death risk. The telomere length from all cell types except NK-cells reached statistical significance [Fig. 5] [Supplementary Data 13], indicating the effect of the telomere length is independent of the effect of age in all-death prediction.

## Discussion

This work presents the largest study to date on telomere length variation in a population cohort based on cross-sectional Flow-FISH^33^ measurements. We measured telomere lengths in six immune-related cell types from 1046 participants from the northern Netherlands LLD cohort^32^. We investigated the effect of genetics and 90 phenotypes, including parental factors and a wide range of general and environment factors, on telomere length. This identified the most important factors that influence telomere length and, based on our findings, we propose potential mechanisms of action for some of these factors. Finally, we identified cell-type-specific transcriptional modules related with immunosenescence that might explain the downstream effects of telomere length attrition.

Previous studies of telomere length variation in population cohorts have mainly relied on classical terminal restriction fragment measurements^77^, qPCR-based methods^78,79^ or computational methods^16^ to measure telomere length. In the current study, we used Flow-FISH in a large epidemiological setting. Flow-FISH allows accurate measurements of the average telomere length in different cell types^80^. This allowed us to observe the distribution of telomere length in different cell types in addition to the more commonly studied telomere length of total leukocyte population^15,18,19^. Our observations reveal a similar trend of telomere length decrease with age and a high correlation in telomere length between different cell types (Pearson correlation’s rho minimum 0.70, mean 0.81, maximum 0.94). These finding overlap with previously published between-tissue telomere length correlation ranges (leukocyte, skin, skeletal muscle and subcutaneous fat, Pearson's rho from 0.76 to 0.88)^81^ but show a smaller range than those reported in post-mortem tissues (highest Pearson’s rho = 0.40 between transverse and sigmoid colon)^20^. Nevertheless, it must be noted that measurements were carried out using Southern blot in ref. ^81^, whereas the measurements in ref. ^20^ where Luminex-based. These findings agree with previous studies that indicated that despite having different telomere lengths in adults, the overall effect of age on telomere length is similar in different cell types^81^. Additionally, our results show high reproducibility with respect to a previous Flow-FISH population study in a North American cohort^9^.

The average telomere length in each cell type is a complex phenotype that reflects cell-type-specific, genetic, intrinsic and environmental effects. Many previous studies have tried to estimate the role of genetics in explaining the variation of telomere length in humans^18^, producing a wide range of estimates. These differences suggest there are potential environmental effects confounding the heritability estimates. For instance, sibling-based studies^17,38,82^ have the disadvantage of not being able to distinguish between genetics and early life or prenatal exposures. In our study, which more closely resembles approaches that estimate “chip-based” heritability in unrelated individuals^83,84^, we estimated narrow sense heritability in six different cell types based on a mixed model approach using sample-to-sample genotype kinship estimations (GREML)^40^. One advantage of this study is that LLD participants were specifically selected to have a common ancestry but to not be highly related among each other, meaning that the GREML estimation should not be biased by those factors. After accounting for age and sex, our GREML estimation indicates a median heritability of 40%, which is in the same range as previously reported^85^. However, our estimate does not include the X or Y chromosomes, which might play a role in telomere heritability^86^. In addition, the relatively small sample size of our study (~1000) hampers the accuracy of our estimation, and we observe large standard error intervals. Nevertheless, the heritability levels that we find are indicative of a large amount of telomere variability being caused by environmental factors, which contrasts with other studies that identified extremely high telomere heritability (0.7^19^, 0.78^82^, 0.81^87^). In our GWAS analysis, we identified one intronic SNP in the TERT gene that was associated at 5 × 10^−8^ and in moderate LD (r^2^ = 0.47) with previously reported associations in this locus. The GWAS analysis in our cohort was limited by our relatively small sample size (911 individuals), but PRS scores from a recent large telomere GWAS^15^ indicate strong association of previously identified genetic variants with telomere length of all cell types.

Despite the on-going efforts to understand the effect of genetics on telomere length^12–16^, a large fraction of unexplained variability remains, which may be attributed to environment. We therefore investigated the relation of 90 phenotypes with telomere length. This analysis pointed to a role for parental phenotypes in the telomere variations in their children. Higher paternal age has previously been associated with longer telomere lengths in humans^19^. Our results agree with this but also identify a maternal age effect on telomere length. Given the high correlation between paternal and maternal age (*r* = 0.77), the maternal age effect we observe might be confounded by paternal age. It is known that sperm telomere length increases with age, whereas telomere length in somatic tissues, including leukocytes, diminishes with age^88,89^ providing a potential explanation for longer leukocyte telomere length in offspring of older fathers. This effect might even be additive over consecutive generations^90^. In addition to parental age, we identified a novel telomere association with parental habits in which parental smoking was negatively associated with telomere length. This effect could either be prenatal or early life.

Additionally, even with the possibility of being caused by common genetic architecture, parental effects in offspring are more plausibly caused by a mechanism other than genetics, such as epigenetic modifications. We, therefore, tested whether methylation might mediate the effect of parental phenotypes such as age and smoking on telomere length in their children. The effect of maternal age, and not paternal age, on telomere length was mediated through methylation of several genes, suggesting that the maternal age effect is not completely confounded by paternal age. Here, we highlighted the mediation of telomere length in NK-cells via the methylation of *SOX11*. It is not clear how *SOX11* methylation may lead to telomere shortening, as *SOX11* is mostly expressed during embryonic development. Previous studies have shown that promoter hypermethylation of *SOX11* inhibits *SOX11* expression in cancer cells^91^. In addition, several studies have shown an anti-senescent effect of *SOX4*, which belongs to the same *SOXC* group as *SOX11*^92^. It is important to stress, however, that this is a proposed mechanism, and that experimental validation will be needed to resolve the nature of such associations.

Apart from parental factors, we identified that women have longer telomeres than men, as previously identified^86,93^. In addition, BMI was associated with shorter telomeres^20,94^. However, we did not observe other previously reported associations, such as with participant’s smoking habits^20,95^. Combining all identified intrinsic, parental and genetic factors other than age with significant effects at least in two cell types, we estimate that only 10%, on average, of the observed telomere length variability can be explained, with only about 3.7% attributable to genetics (without accounting for the genetics factors that cause sex differences). These findings showcase a greater impact of environmental and intrinsic effects than genetics on telomere length variation. Nevertheless, it is worth noting that the model applied in this study does not consider interactions between the different layers of information, where genetics could potentially impact the effects of the other environmental features. In addition, we are only considering highly associated additive SNPs and no other possible epistatic relationships, which means we are probably only setting a lower bound on the amount of variability that we can account for.

Our single-cell transcriptomic analyses identified a set of 97 unique genes that are significantly associated with telomere length across T-cell types. Three of them (*DNAJA1, EEF1A1* and *RPL29*) were previously revealed as telomere binding proteins^51^, indicating that our approach captures genes directly involved in telomere length dynamics. Moreover, our study provides additional insight into the direction of effect. For example, we found *RPL29* to be negatively associated with telomere length, which is in line with previous studies describing accumulation of RPL29 as a biomarker of senescence^73^. Looking at the broader context, we observed functional enrichment of genes involved in translation and the NMD pathway within the gene set negatively associated with telomere length in T-cells, which might have important physiological consequences.

Even though some of the telomere length–associated genes were located near the telomere ends (i.e., 12 and 9 DE genes that fall < 4 Mb and between 4–10 Mb from the telomere ends, respectively), in general, our differential expression findings could not be explained by previously described mechanisms (TPE^26,27^ and TPE-OLD^28^). This suggests that there are other mechanisms by which telomere length regulates gene expression, or vice versa^51^. Follow-up experimental perturbation studies will be required to make this distinction, for example by inducing telomere shortening (replicative senescence) followed by gene expression analysis or by inducing gene knockdowns followed by telomere length measurements.

Finally, by making use of longitudinal data on participant survival, we were able to replicate a negative association between telomere length and all-cause mortality^96^, although the small number of deceased participants (11) hampers the wider interpretation of these results. Further studies of Lifelines participants will clarify this predicted relationship^97^.

## Methods

### Lifelines deep cohort

Lifelines is a multi-disciplinary prospective population-based cohort study examining, in a unique three-generation design, the health and health-related behaviours of 167,729 persons living in the North of the Netherlands. It employs a broad range of investigative procedures to assess the biomedical, socio-demographic, behavioural, physical and psychological factors that contribute to the health and disease of the general population, with a special focus on multi-morbidity and complex genetics^41^. The Lifelines study was approved by the medical ethical committee from the University Medical Centre Groningen (METc number: 2017/152). We collected data from the subcohort Lifelines DEEP (LLD, *n* = 1057, 57.6% female, mean age (including months) 43.9 years [range 18–81.4 years])^32^. Extensive information on demographics, health and lifestyle factors including smoking and diet was collected via detailed questionnaires. Mean BMI of participants was 25.1 [range 15.8‒44.9]. Common age-related diseases within the cohort included hypertension (23% of 841 participants with information), type 2 diabetes (1.3% of 1039 participants with information) and hypercholesterolemia (14% of 900 participants with information). In the cohort, 20% of individuals smoked currently, 48% smoked for at least 1 year and 37% had mothers and 65% had fathers who smoked.

Genome-wide genotyping data was generated as described in ref. ^32^. Genotype data processing was done as described in ref. ^98^. Briefly, microarray data were generated on CytoSNP and ImmunoSNP and then processed on the Michigan Imputation Server^99^ to perform phasing using SHAPEIT and imputation using HRC version R1 as reference^100^. We excluded SNPs with imputation quality r^2^ < 0.5, minor allele frequency (MAF) < 0.05, call rate < 0.95 and Hardy–Weinberg equilibrium test *p* < 1 × 10^−6^, which resulted in 5,327,634 SNPs used in subsequent analyses. Genotype data was available for 911 samples with non-missing telomere measurements.

Genome-wide methylation data generation for this cohort was described previously^101^. Briefly, the EZ DNA Methylation kit (Zymo Research) was used to bisulfite-modify 500 ng of genomic DNA, which was hybridised on Illumina 450 K array. Methylation probes were remapped to ensure their correct genomic location, and probes with known SNPs at the single base extension site or CpG site were removed. Next, data were processed using a pipeline described by Tost and Touleimat^102^. We used DASEN-normalised data with subsequent quantile normalisation and probe scaling applied. Methylation data for 418,499 probes was available for 651 samples with telomere measurements in at least one cell type.

### Flow-FISH Telomere length measurement

Telomere length measurements using automated multicolour flow-fluorescence in situ hybridisation (Flow-FISH) were performed as described^33^. Briefly, white blood cells were isolated by osmotic lysis of erythrocytes in whole blood using NH_4_Cl. White blood cells were then mixed with bovine thymocytes of known telomere length (which served as an internal control), denatured in formamide at 87 °C, hybridised with a fluorescein-conjugated (CCCTAA)3 peptide nucleic acid (PNA) probe specific for telomere repeats and counterstained with LDS751 DNA dye. The fluorescence intensity in granulocytes, total lymphocytes and lymphocyte subsets defined by labelled antibodies specific for CD20, CD45RA and CD57 relative to internal control cells and unstained controls was measured by flow cytometry to calculate the median telomere length from duplicate measurements.

Out of a total of 1388 participants, we could not measure the telomere length in any cell type in 207. In addition, granulocytes could not be measured in 109 participants, B-cells could not be measured in nine participants and NK-cells could not be measured in 17 participants (all of these were different participants). We decided to remove all participants with at least one missing cell type, which resulted in a final subset of 1046 participants.

### sjTREC measurement

DNA from whole blood was isolated using the conventional protocol with Proteinase K digestion followed by phenol extraction and isopropanol precipitation. Next, we performed a TaqMan quantitative real-time PCR approach to quantify sjTREC expression (signal joint excision circles produced during T-cell development) using ViiA™ 7 Real-Time PCR System (Life technologies). TaqMan qPCR was performed on 75–100 ng DNA in a 12 μl reaction mixture containing 18 µM of each primer for sjTREC (5′‐TCGTGAGAACGGTGAATGAAG‐3′ and 5′‐CCATGCTGACACCTCTGGTT‐3′) and for albumin as housekeeping gene (5′‐TGAACAGGCGACCATGCTT‐3′ and 5′‐CTCTCCTTCTCAGAAAGTGTGCATAT‐3′) and 5 µM of hydrolysis sjTREC probe 5′‐(FAM) CACGGTGATGCATAGGCACCTGC‐3′ (TAMRA) and albumin probe 5′‐(FAM) TGCTGAAACATTCACCTTCCATGCAGA ‐3′ (TAMRA). PCR runs started with incubation at 50 °C for 2 min, then at 95 °C for 15 min, followed by 45 cycles of denaturation at 95 °C for 15 s and annealing/elongation at 60 °C for 30 s. All reactions were carried out in duplicate per sample using sjTREC primers and sjTREC probe as well as albumin as a single-copy albumin gene to normalise the results, taking into account the amount of input DNA. During PCR, the amplification mediated the cleavage of the probes, which contain a quencher (TAMRA) and a reporter (FAM) dye. This, in turn, leads to the separation of the quencher from the reporter, thereby inducing fluorescence of the reporter dye. The expression of target (sjTREC and albumin) in analysed samples was established by measuring the threshold cycle (CT), defined as the cycle number at which the fluorescence generated by cleavage of the probe passes a fixed threshold above baseline. We calculated the standard deviation between the duplicates, and the results were accepted for further analysis when the standard deviation was ≤1. Next, we considered the average for all the duplicates with standard deviation ≤1, and the normalised sjTREC expression in each sample was calculated as a difference between average CT values of albumin and average sjTREC CT values.

### Definition of telomere fast and slow agers and comparison between biological ageing methods

Telomere lengths were used to identify participants with above or below average telomere length. To do so, we fitted a linear model to each cell type’s telomere length using age, including months, as regressor. We then identified participants under one standard deviation of their predicted value. We considered all participants with at least two cell types passing the one standard deviation threshold to be fast or slow agers. Participants identified with some cell types above one standard deviation and some below were excluded. Thus, we defined two groups of participants: fast and the slow agers. We then performed a logistic regression using either sjTREC or methylation age as a regressor explaining this binary category (fast agers or slow agers) while controlling for age, sex and cell counts.

### GREML heritability estimation

We used the GCTA^40^ software for narrow sense heritability estimation. We used the microarray SNP data available in LLD to calculate a genetic relationship matrix (GRM) using variants with a MAF > 1% (GCTA–make-grm). Next, we used the estimated GRM to calculate the amount of variance explained by the random effect of genetics in a linear mixed model (GCTA–reml) while accounting for sex and age as fixed-effect covariates.

### GWAS of telomere length

Using a previously described pipeline^103^, we performed an association analysis of telomere length of each cell type with genome-wide SNP genotype data by calculating the Spearman correlation coefficient between telomere length measurements corrected for age and sex and SNP genotype dosages. We corrected the results for multiple testing by permuting genotype labels 10 times to create a null distribution that was used to control FDR at 0.05.

### Polygenic risk score calculation

The contribution of genetics was calculated using a PRS created using 20 independent genome-wide significant loci reported a large European-descent telomere GWAS study^15^. A weighted PRS was calculated for each LLD participant using PLINK 1.9–score sum function^104^. The PRS was used as a regressor using chronological age as covariate and telomere length of each cell type as dependent variables of six different linear models.

### Phenotype correlations

We preselected 90 phenotypes for correlation with telomere length based on the relevance and sample size of each phenotype. We used telomere length as the dependent variable while the phenotype was standardized to unit variance and used as the explanatory variable. Linear models were fitted in R (v4.0.1) by ordinary least squares (OLS). We fitted all models while accounting for the effect of age and sex. We then fitted a second model that also accounted for the effect of different blood cell counts available per sample, namely granulocytes (basophils, eosinophils and neutrophils), erythrocytes, lymphocytes, monocytes and thrombocytes measured per plasma litre. We computed FDR from all *P* values using the Benjamini-Hochberg procedure^105^ as implemented in R base functions.

For some specific diabetes-related and BMI-related phenotypes, such as ‘Waist circumference in cm’ and ‘Body Mass Index (kg/M^2^)’, we included paternal and maternal age and parental smoking habits as additional covariates in the model discussed above. At the same time, to disentangle maternal and paternal age effects, we applied a linear model including covariates (age, sex, cell counts) and both phenotypes, using each telomere length as dependent variable. We included an l1 penalisation (Ridge regression) using glmnet in R^106^ to shrink the estimates of superfluous covariates. The strength of the regularisation parameter (λ) was estimated by a 10-fold cross-validation.

To identify possible confounders in BMI-related phenotypes, we performed correlations between BMI and parental smoking and parental age using an additional 10,000 participants from the Lifelines study population^41^.

### Assessment of total variance explained by phenotypes

To assess the total variability explained by each layer of information, we selected all phenotypes found significantly associated with at least two telomere lengths under an FDR of 0.05. First, we regressed out the effect of chronological age in telomere length. Next, we fitted three nested models with intrinsic host factors: one adding sex, a second adding cell counts and a third adding waist circumference and BMI. These were fitted using a lasso shrinkage model (using glmnet in R^106^) to explain the residuals of telomere length after removing the chronological age effect. A fourth model added the parental phenotypes age and smoking to the intrinsic factors model. The final model added the PRS (see *Polygenic Risk Score calculation*) on top of the parental model. We computed the variance explained (R^2^) by each model and the ΔR^2^ of the variability gained by each layer of information introduced.

### Methylation association analyses

We performed a EWAS of all probes with parental phenotypes using OLS in R. The following five phenotypes were used: age of mother and father at participant’s birth, maternal smoking during childhood, maternal smoking during pregnancy and paternal smoking during childhood, all while correcting for age, sex and cell counts as covariates. Bonferroni multiple testing correction was applied to correct for 5 × 418,499 = 2,092,495 tests. Next, we assessed the association of methylation and telomere length using a similar linear model, adjusting for age, sex and cell counts.

### Mediation analysis

To test if the parental phenotype effect on telomere length may be mediated by methylation of certain probes, we performed a mediation analysis. For this we selected triplets of parental phenotypes (independent variable), methylation probes (mediator) and telomere length (dependent variable) using the following criteria: We required that a parental phenotype was associated with the methylation probe with FDR < 0.05, that methylation at this probe was associated with telomere length at a *p* < 0.05 and that the parental phenotype was associated with telomere length at a *p* < 0.05. Next, we estimated mediation using the mediation R package^107^, adding age, sex and cell counts as covariates to all models. We performed a Benjamini-Hochberg multiple testing correction on the average causal mediation effect (ACME) *p* value and required that the FDR < 0.05 and the average direct effect (ADE) *p* value was >0.05 (meaning that the parental phenotype effect is mostly mediated by methylation). In addition, we investigated a slightly different situation where methylation and telomere length are swapped. Here, we checked if the parental phenotype effect on methylation is mediated by telomere length. While the real causality directions are very complex and often contain loops, and as a consequence these swapped ACME *P* values are often <0.05, we required that a swapped ACME *p* value be larger than the main original ACME *p* value and that the mediated proportion of the effect for the original direction be larger than for the swapped scenario.

### Differential expression analysis at the single-cell level

To study gene expression changes with telomere length at the single-cell level, we used a subset of previously processed scRNA-seq data^49^ on unstimulated peripheral blood mononuclear cells from 62 LLD donors for whom we collected Flow-FISH telomere length data for at least one cell type in the current study. This scRNA-seq data was generated 5 years after collection of the Flow-FISH telomere length data. In short, scRNA-seq data was generated using the 10X Chromium Single Cell 3’ V2 chemistry and libraries were 150 bp paired-end sequenced on Illumina’s NovaSeq6000. The Cellranger v3.0.2 pipeline was used with default parameters to demultiplex, generate FASTQ files, map reads to the GRCh37 reference genome and generate a unique molecular identifier (UMI)-corrected count matrix per cell^108^. After quality control, 54,373 cells remained. In the original dataset, k-nearest neighbour clustering was used to cluster the cells. We then performed automated cell type classification using Azimuth to annotate the cells^50^. In detail, we conducted a supervised analysis guided by a reference dataset to enumerate cell types that would be challenging to define with an unsupervised approach. Thus, we mapped our scRNA-seq query dataset to a recently published CITE-seq reference dataset of 162,000 PBMC measured with 228 antibodies^50^. For this process, we used a set of specific functions from the Seurat R package v4.0.0^50,109^. First, we normalised the reference dataset using the SCTransform function. Then, we found anchors between reference and query using a precomputed supervised PCA transformation through the FindTransferAnchors function. Afterwards, we transferred cell type labels and protein data from the reference to the query. We also projected the query data onto the UMAP structure of the reference. For these two last steps, we used the FindTransferAnchors function. Finally, the high resolution cell-type-annotations predicted by Azimuth (celltype.l2) were combined in such a way to more closely reflect the resolution of the Flow-FISH annotations (i.e., naïve and memory CD4T and CD8T cells, NK- and B-cells) (Supplementary Data 8).

Telomere length DE analyses were conducted using MAST v1.16.0^110^ at single-cell resolution (sc-DEA) by selecting the matched telomere length measurement and gene expression level for each of these cell types. As a first approach (I), we performed an independent telomere length sc-DEA for each of the previously defined cell types (i.e., naïve and memory CD4T and CD8T cells, NK- and B-cells). We then conducted a second approach (II) in which we combined multiple (sub)cell types together in the same analysis (i.e., we performed five different sc-DEA combining: all CD4T, CD8T, naïve T, or memory T and T-cells) while controlling for cell type annotation (Supplementary Data 8). As our first approach had indicated that we had insufficient cells to detect telomere length–associated DE genes in B- and NK-cells, we did not re-analyse these cells using our second approach. This strategy allowed us to increase the number of cells per donor per analysis and thereby increase the power to detect potential effects. In both cases, as input, we used the log-normalised and scaled expression counts from those genes expressed in at least 10% of the cells. MAST uses a two-part generalised linear model, specifically a Hurdle model, on zero-inflated continuous data in which the zero process is modelled as a logistic regression and the continuous process is modelled as a linear regression. To accommodate the complex experimental design while controlling for covariates, including both biological variables (sex, age, donor and cell type) and technical factors (cellular detection rate (CDR) and 10x lane/experimental batch), we fitted a general linear mixed model (glmer) of the form:

I $${{{{{{\rm{Expr}}}}}}}_{{{{{{\rm{i}}}}}}} \sim {{{{{{\rm{telomere \,length}}}}}}}_{{{{{{\rm{i}}}}}}}+{{{{{{\rm{CDR}}}}}}}_{{{{{{\rm{i}}}}}}}+{{{{{\rm{sex}}}}}}+{{{{{\rm{age}}}}}}+(1|{{{{{\rm{donor}}}}}})+(1|{{{{{\rm{lane}}}}}})$$

II $${{{{{{\rm{Expr}}}}}}}_{{{{{{\rm{i}}}}}}} \sim {{{{{{\rm{telomere \,length}}}}}}}_{{{{{{\rm{i}}}}}}}+{{{{{{\rm{CDR}}}}}}}_{{{{{{\rm{i}}}}}}}+{{{{{\rm{sex}}}}}}+{{{{{\rm{age}}}}}}+{{{{{\rm{celltype}}}}}}.{{{{{\rm{l}}}}}}{2}_{{{{{\rm{i}}}}}}+(1|{{{{{\rm{donor}}}}}})+(1|{{{{{\rm{lane}}}}}})$$

where Expr_i_ is the log-normalised, scaled expression of the gene being tested in cell i; telomere length_i_ is the telomere length measurement using Flow-FISH in the tested cell type; CDR_i_ is the fraction of genes that are detectably expressed in each cell; celltype.l2_i_ is the high resolution cell type annotation predicted by Azimuth; sex and age are phenotypic variables from the donors; donor is the donor LLD id and lane is the experimental batch, here being the lane of a 10X chip. First, we used the zlm function to fit a glmer (method = ‘glmer’, ebayes = FALSE, fitArgsD = list, nAGQ = 0, all other parameters set to default). Some of the gene models failed to converge when considering random effects (i.e., donor and lane), which resulted in NA estimated coefficients. To mitigate this convergence issue, nAGQ = 0 was passed to the fitting function. We then used the summary function to perform a likelihood ratio test (LRT) to test for differences when we drop the telomere length factor (doLRT = telomere length, fitArgsD = list, nAGQ = 0, all other parameters set to default).

Finally, genes were considered to be DE with telomere length when gene expression change was significant at an FDR < 0.05. The EnhancedVolcano function from EnhancedVolcano R package v1.8.0 was used to visualise the gene’s significance and log2FC. The pheatmap function from pheatmap R package v1.0.12 was used to visualise the expression pattern (log-normalised counts), clustering and annotation of the set of 97 unique DE among T-cells. For visualisation purposes, we down-sampled each of the Azimuth’s predicted T (sub) cell types to 100. To distinguish the four main expression patterns, the argument cutree_rows = 4 was set when using the pheatmap function. The pheatmap function was also used to visualise the LFC per telomere length unit among the set of 97 unique DE genes identified in T, CD4T and CD8T cells.

### Literature validation of single-cell analysis

To validate our sc-DEA findings, we compared our results to four biologically related studies that: (i) revealed novel telomere proteins using in vivo cross-linking, tandem affinity purification and label-free quantitative LC-FTICR-MS^51^, (ii) identified a set of genes associated with ageing in whole blood using bulk RNA-seq after correcting for cell type composition^53^, (iii) created the GenAge human database as part of the HAGR databases composed of 307 human ageing-related genes^54^ and (iv) defined an association between telomere length and methylated cytosine levels for both blood and EBV-transformed cell-line DNA samples^29^. To this end, we overlapped the set of 97 unique genes we found to be DE with telomere length against these four previously reported lists of genes and performed a two-sided Fisher’s exact test to assess whether these overlaps were significant. We could not test the overlap with ref. ^29^ since multiple methylated CpG sites are linked to the same gene. For the tested overlaps, as our background list of genes, we gather the union of all tested genes in T-, CD4T and CD8T cells. As each study’s background list of genes, we considered all the tested genes in ref. ^53^ and all the protein-coding genes (using Gencode v26, GRCh38 annotation) in refs. ^51,54^.

### Single cell functional enrichment analysis

We performed a functional enrichment analysis through an over-representation analysis using WebGestalt (WEB-based GEne SeT AnaLysis Toolkit)^111^. As the input gene list, we used the 44 and 91 DE genes we identified in CD4T and all T-cells, respectively, split by their direction of effect (i.e., positively or negatively associated with telomere length). As the background gene set, we used the 2426 and 2427 expressed genes that were tested in the sc-DEA of the CD4T or all T-cells, respectively. As functional databases, we used two different pathway databases: KEGG and Reactome. Advanced default parameters were used (minimum and maximum number of genes for a category: 5 and 2000, multiple test adjustment: BH, significance level: FDR ≤ 0.05, number of categories expected from set cover: 10, redundancy reduction: affinity propagation and weighted set cover).

### Differentially expressed genes telomere distance analysis

We compared the shortest distance to the telomere of DE genes compared to non-DE genes in CD4T cells and T-cells. Gene locations were retrieved using the human Ensembl BioMart dataset GRCh38.p13^112^. Chromosome length was used as a proxy for telomere location. We took the minimum between the start of the gene position and the distance between the end of the gene and the end of the chromosome as the distance from the gene to the telomere. We then performed a one-sided Fisher’s exact test to examine whether there was an enrichment of genes located at subtelomeric regions in the DE sets in comparison to background non-DE sets. We performed this analysis in positively and negatively telomere-associated genes separately and in each of the two cell types. To overcome a possible confounder effect of chromosome length, we subsampled with no replacement genes from the non-DE gene set while keeping the same chromosome proportions observed in the DE gene set, and again performed a Fisher’s exact test.

### Survival analysis

We obtained survival information for 1044 participants and ultimately the dates on which 11 participants had died. We measured the number of survival days since blood was donated. The R ‘survival’ package was used to perform a Cox regression analysis using survival (days) as dependent variable and telomere length as explanatory variable, while controlling for age, sex and blood cell counts. We tested the proportional hazards assumption, both of telomere length as a covariate and for the whole model (cox.zph function), using a *p* value cut-off of 0.05.

### Reporting summary

Further information on research design is available in the Nature Research Reporting Summary linked to this article.

## Supplementary information

Supplementary Information

Description of Additional Supplementary Files

Supplementary Data 1-13

Reporting Summary

## Data Availability

The data here presented belongs to Lifelines. Lifelines are specifically organized to make assessment results available for (re)use by third parties. The biological ageing data used here, including sjTRECs expression, methylation age predictions, telomere length measurements, genetics, methylation and phenotypic data can be requested through Lifelines. A research proposal must be submitted for evaluation by the Lifelines Research Office. • Phenotypic data: Researchers must submit a data order (i.e., a selection of variables) and research proposal in the Lifelines online catalogue. • Omics data: Omics data are stored in the UMCG HPC. Omics data cannot be ordered using the Lifelines online catalogue, but are made accessible in full to researchers with an approved requst. With uniquely made linkage files the researcher can link an order of phenotypic data to omics data, if requested. In addition to this, processed (de-anonymized) scRNA-seq data, including a text file that links each cell barcode to its respective individual, is available at the European Genome-Phenome Archive (EGA), under accession number: *EGAS00001005376*. A toy-example dataset is available to run the single-cell analysis is available in the MOLGENIS^113^ cloud and can be access from the following link: https://downloads.molgeniscloud.org/downloads/combio_andreu_2022/ GWAS summary statistics generated in this study are available at GWAS catalogue, under the accession numbers: Granulocytes - *GCST90101887*; lymphocytes - *GCST90101888*; naïve T-cells - *GCST90101889*; memory T-cells - *GCST90101890*; B-cells - *GCST90101891*; NK-cells - *GCST90101892*.

## Code availability

Analysis scripts are publicly available at: https://github.com/GRONINGEN-MICROBIOME-CENTRE/Groningen-Microbiome/tree/master/Projects/Telomere_analysis.
