# Supplementary material for: Genetic, parental and lifestyle factors influence telomere length
Source: Commun Biol. 2022 Jun 9;5:565. doi: 10.1038/s42003-022-03521-7 (PMC9184499; doi:10.1038/s42003-022-03521-7)
Supplement: Supplementary file 1 — Supplementary Information [file 42003_2022_3521_MOESM1_ESM.pdf]

Supplementary Materials

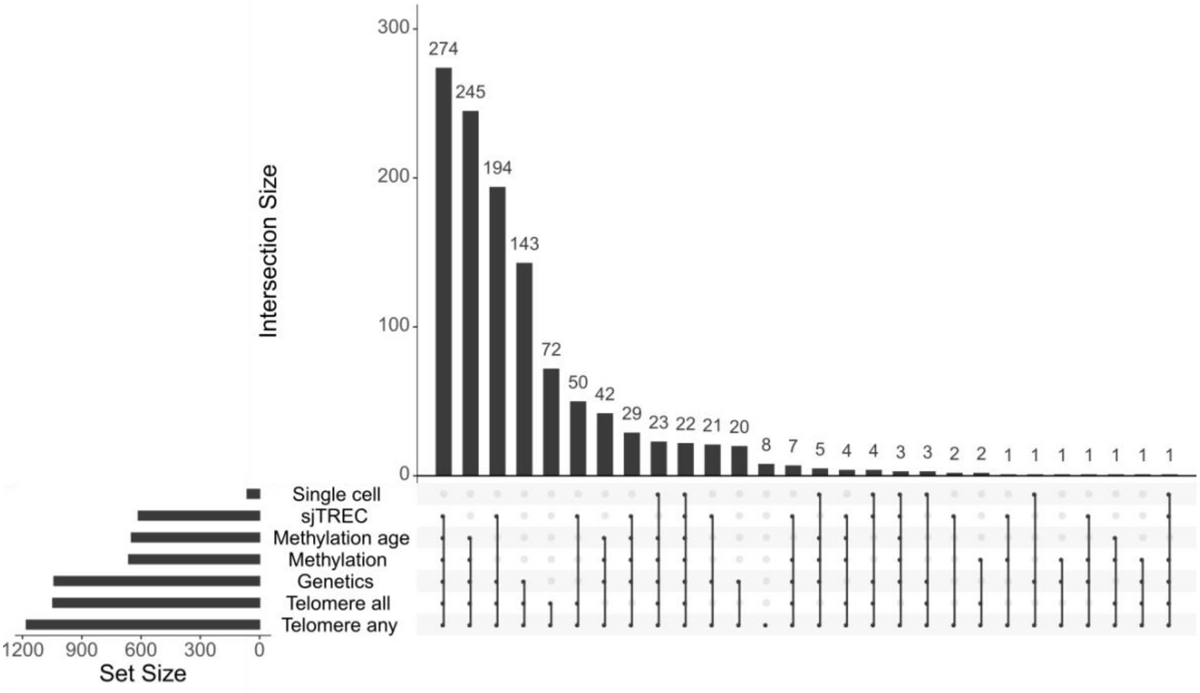

Supplementary Figure 1. **Phenotype markers in the LLD cohort**. Representation of omics data used in this work. Horizontal bars indicate the number of Lifelines participants who had available information for the specific omics layer. Vertical lines indicate the number of samples in the intersection of the different layers and not seen in any other layer. Single cell (62) indicates the number of samples with single-cell RNA-seq data. sjTREC (613) indicates the number of people with qPCR measurements of sjTRECs. Methylation age (648) is a subset of participants where age was predicting using the methylation probes. Methylation (662) indicates the number of participants with available methylation arrays. Genetics (1,040) is the number of participants with available microarray data. Telomere any (1,180) are the number of participants with at least one cell population where telomere length was measured. Telomere all (1,046) are the number of participants with telomere lengths for all six cell populations available.

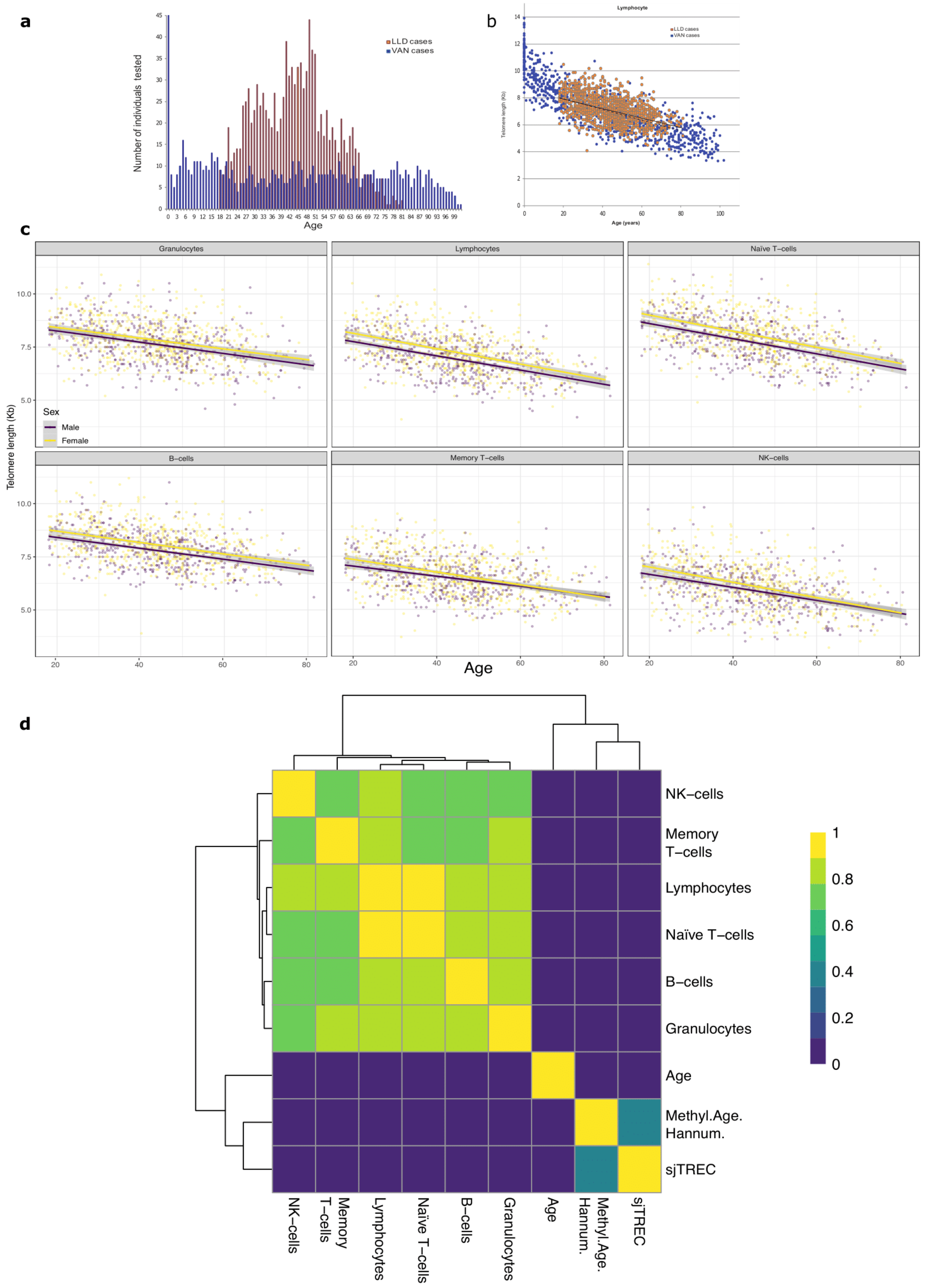

Supplementary Figure 2. **Sample cohort descriptions and measurements comparison. a.** Number of study participants per age year in the Canadian study (VAN, blue, total n = 835, ages 18–81, n = 490) and the LLD

study (LLD, orange, total n = 1049). **b.** Total lymphocyte cell type telomere length measurements (Kb) comparison between studies. Canadian cohort in blue. LLD cohort in orange. Each point represents an individual. 50th percentiles of distribution are shown for each cohort and are consistent with one another (Canadian cohort light blue boundaries and dashed line, LLD cohort red boundaries and full line). The higher variability between models at older age is attributed to differences in study design and recruitment. **C.** Telomere length decreases with age in six different cell types. Each point represents a participant's telomere length (Kb) in each cell type. Colour shows the sex of the participant. Trend line presents the average telomere length for a given age and sex group. Grey area surrounding trend lines indicates the 95% confidence interval of the trend line. **D.** Correlation (Spearman's rho value) between the absolute value of telomere lengths, Hannum-based methylation age and sjTREC qPCR relative expression residuals after chronological age (Age) regression.

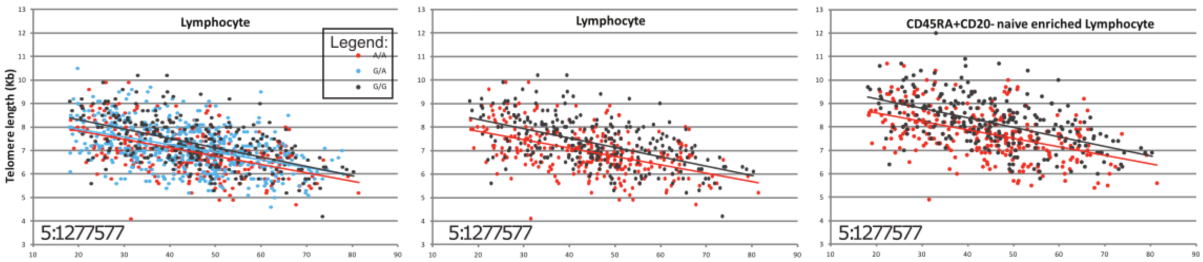

Supplementary Figure 3. Telomerase SNP genotype with significantly different telomere length. *TERT* rs33961405 (chromosome 5:1277577) genotypes and respective linear regression are shown for the lymphocyte cell subset, with homozygous genotypes displayed for the lymphocyte and the CD45RA+CD20-naïve enriched cell type.

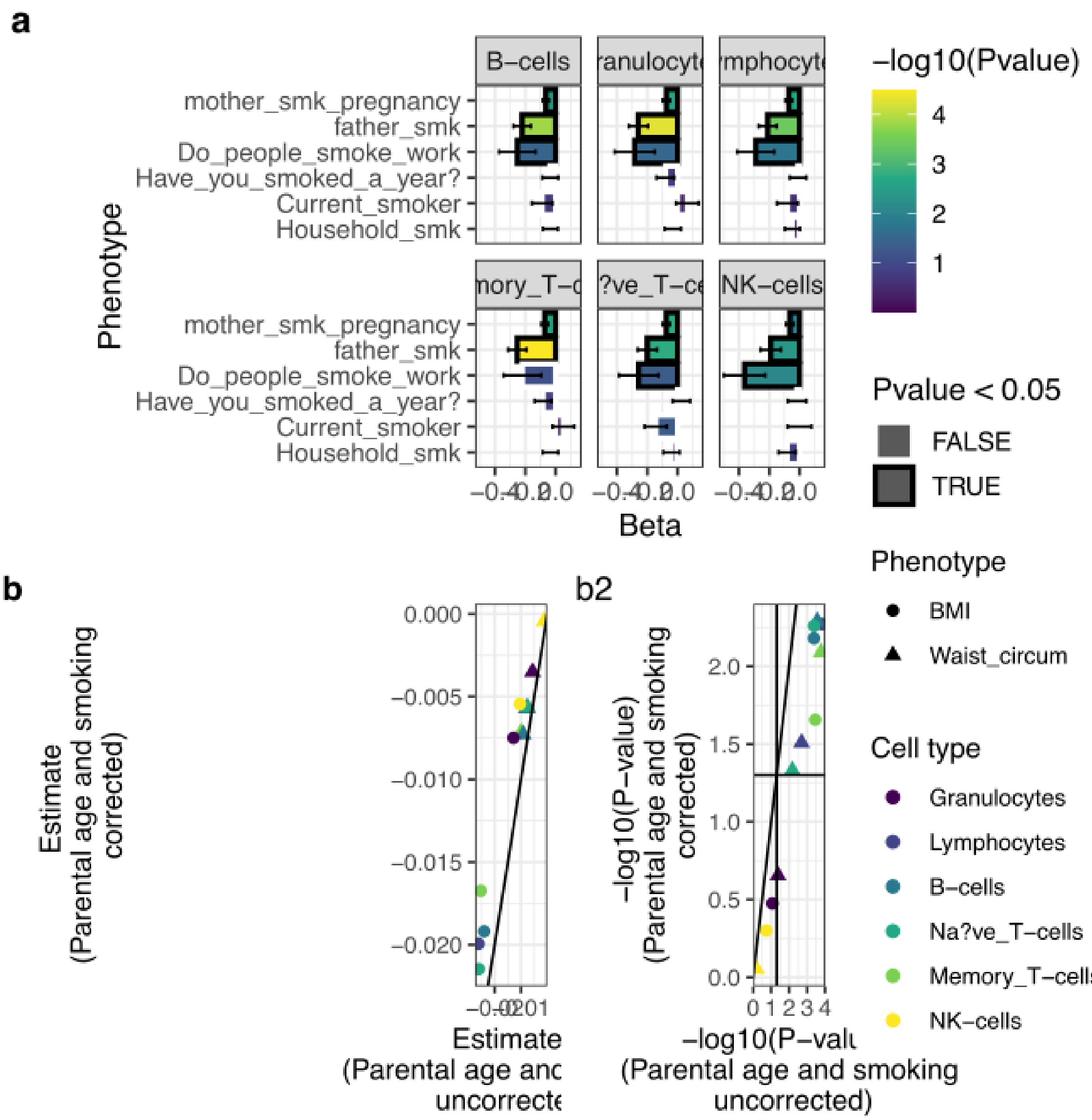

Supplementary Figure 4. **Phenotype exploration. a.** Smoking effects on participant telomere length**.** Length of bar represents the linear model estimate. Colour shows the estimated P-value. Black outline indicates significant associations (nominal P<0.05). Error bars indicate the standard deviation of the estimation. Most phenotypes contain two levels (yes/no), except household_smk, which is a continuous variable of the number of people smoking at the participant's residence, and mother_smk_pregnancy, which is a 4-level factor that includes: mother did not smoke, mother smoked before pregnancy, mother quit/reduced smoking during pregnancy and mother kept smoking regularly during pregnancy. **b.** BMI phenotypes corrected for parental age and smoking status. Left panel shows estimated effect size of BMI (circles) and waist circumference (triangles) on telomere length, while controlling for parental age and smoking status (y-axis) and without correction (x-axis). Associated -log10(P-values) of the estimates are shown in the right panel. Vertical and horizontal lines represent the nominal P-value significance threshold (0.05).

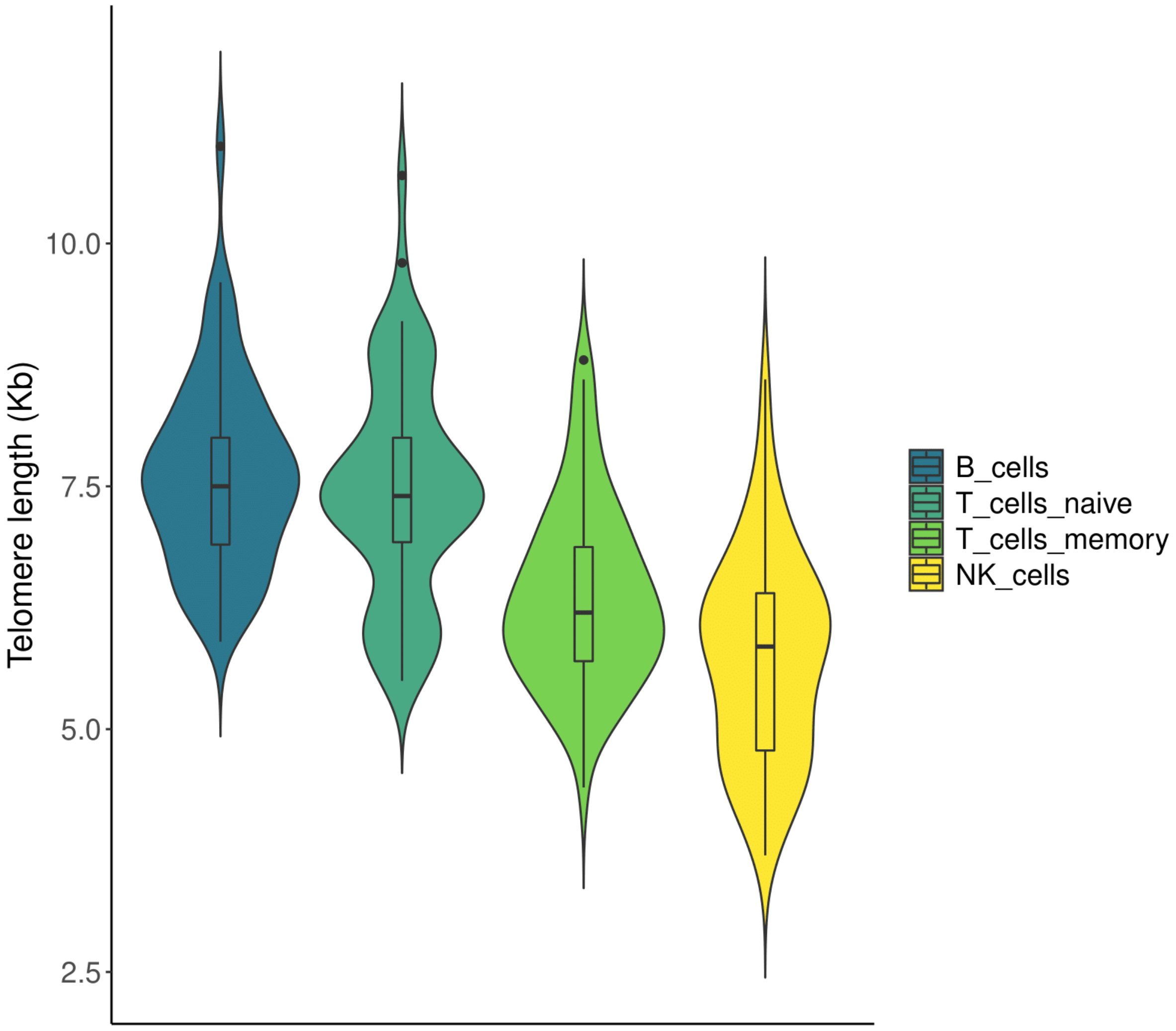

Supplementary Figure 5. **Telomere length in the subpopulations with single-cell expression data.** Distribution of telomere length by cell type in the subset of 62 LLD donors for which both scRNA-seq and Flow-FISH telomere length data was collected. We confirmed that this subset of 62 LLD donors showed similar telomere length distributions to the total study population of 1,046 LLD donors in which telomere length was assessed (Figure 1A).

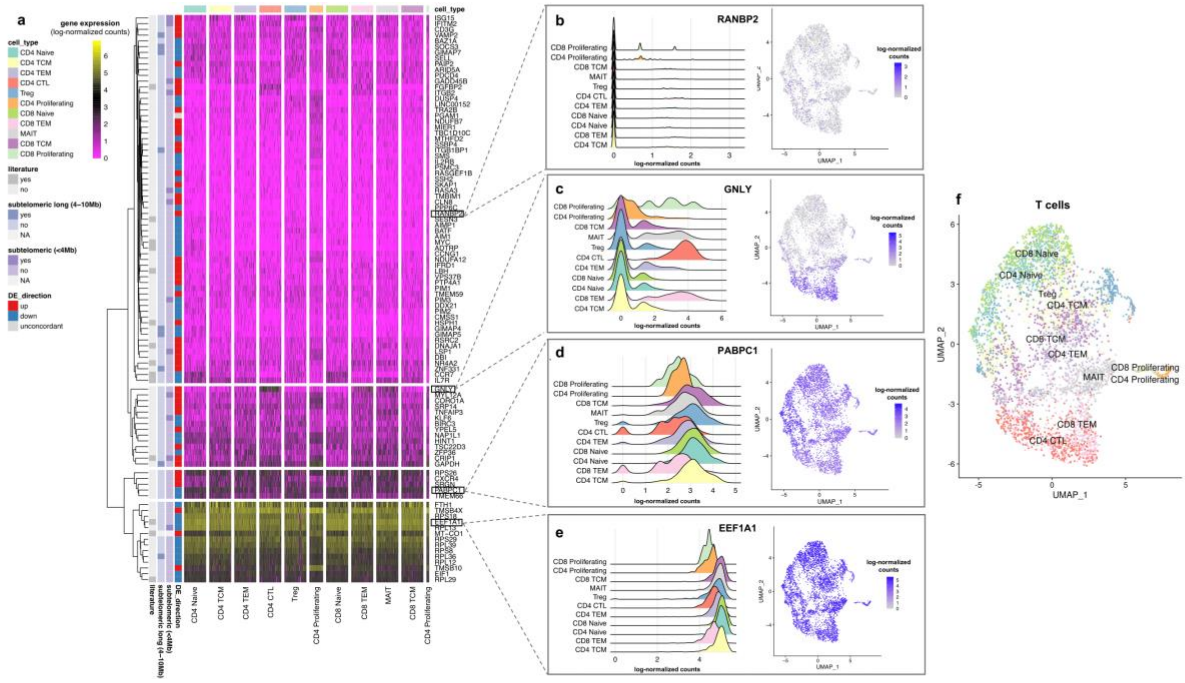

Supplementary Figure 6. **Gene expression pattern of the DEGs obtained through the DEA approach II across Azimuth's predicted T-(sub)cell types. a.** Heatmap of the gene expression (log-normalised counts) pattern of the set of 97 unique DEGs identified in T-, CD4T and CD8T cells. The hierarchical clustering of the genes revealed four main expression patterns. From bottom to top: genes highly and ubiquitously expressed, genes moderately and ubiquitously expressed, genes preferentially expressed in a particular cell type, and genes lowly and ubiquitously expressed. The column annotation bar shows all the cells from the same cell type. The row annotation bars show the DEG direction (DE_direction), whether the DEG is located at the subtelomeric (<4Mb) or subtelomeric long (4-10Mb) region, and if the DEG was previously reported in any of the following studies: Pellegrino-Coppola et al., 2021[53], Tacutu R et al., 2018[54], Buxton JL et al., 2014[29] or Nittis T et al., 2010[51] (literature) (**Supplementary Table 8**). Only one DEG (PGAM1) showed a different direction among T, CD4T and CD8T DEAs (DE_direction = unconcordant). The distance to the telomeres was not calculated for the mitochondrial gene MT-CO1 (subtelomeric (<4Mb) and subtelomeric long (4-10Mb) = NA). For visualisation purposes, we down-sampled each of the cell types to 100 cells. **b-e.** Examples of the four main gene expression patterns described in (**A**). Left: Gene expression (log-normalised counts) distribution plots across T (sub) cell types. Right: T-cells UMAP plots coloured by gene expression (log-normalised counts). **F.** T-cells UMAP plot coloured by Azimuth's predicted T (sub) cell types. In **b-f**, we down-sampled each of the cell types to 500 cells for visualisation purposes.

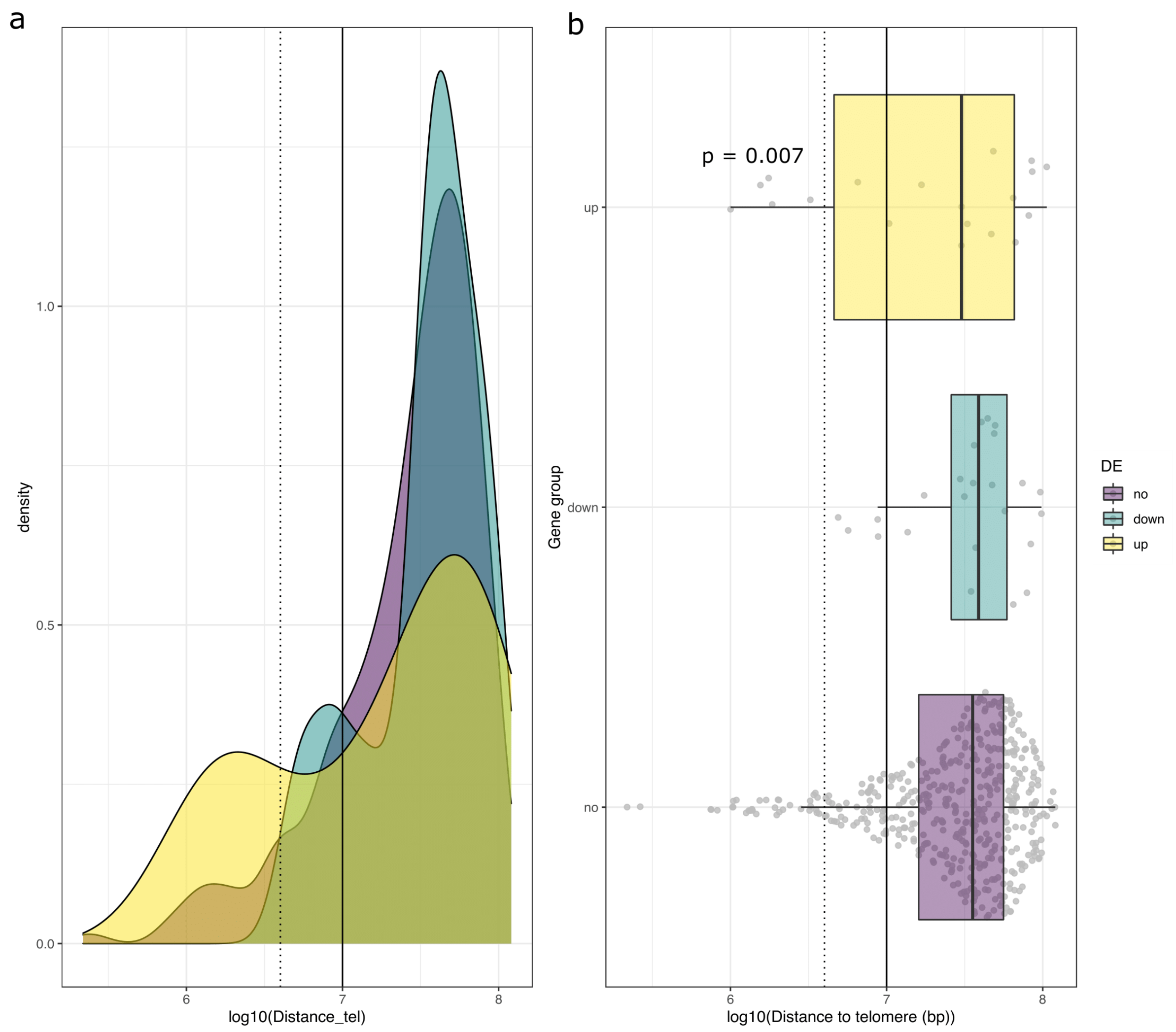

Supplementary Figure 7. Distance to telomere of DE gene sets. Comparison of distance to the telomeres between DE and background genes in CD4 T-cells. **a**. Distribution of distance to telomere of DE genes (downregulated (blue) and upregulated (yellow)) and background (purple). **b**. Tukey boxplot of background genes, genes negatively associated with telomere length and genes positively associated with telomeres. Vertical lines represent subtelomeric region length (dotted, 4Mb) and maximum distance where TPE-OLD has been detected (not dotted, 10Mb).
