# Supplementary material for: Genetic, parental and lifestyle factors influence telomere length
Source: Commun Biol. 2022 Jun 9;5:565. doi: 10.1038/s42003-022-03521-7 (PMC9184499; doi:10.1038/s42003-022-03521-7)
Supplement: Supplementary file 2 — Description of Additional Supplementary Files [file 42003_2022_3521_MOESM2_ESM.pdf]

Description of Additional Supplementary Files

File name: Supplementary Data 1

Description: Descriptive statistics for samples in each data layer.

File name: Supplementary Data 2

Description: Telomere genetic associations - Li et al 2020 replication.

File name: Supplementary Data 3

Description: Telomere genetic associations - Novel GWAS (P<1*10^-5).

File name: Supplementary Data 4

Description: Phenotype overview.

File name: Supplementary Data 5

Description: Phenotype vs Telomere length associations. Age, sex corrected.

File name: Supplementary Data 6

Description: Phenotype vs Telomere length associations. Age, sex, cell count corrected.

File name: Supplementary Data 7

Description: Methylation mediation effect to parental phenotypes associations with telomere length.

File name: Supplementary Data 8

Description: Single cell. Azimuth Cell type classification.

File name: Supplementary Data 9

Description: Single cell. DEA results using method 1.

File name: Supplementary Data 10

Description: Single cell. DEA results using method 2.

File name: Supplementary Data 11

Description: Single cell. Annotation of DEA genes.

File name: Supplementary Data 12

Description: Single cell. Functional enrichment.

File name: Supplementary Data 13

Description: Summary statistics Cox-regression.
