# Supplementary material for: Genetic, parental and lifestyle factors influence telomere length
Source: Commun Biol. 2022 Jun 9;5:565. doi: 10.1038/s42003-022-03521-7 (PMC9184499; doi:10.1038/s42003-022-03521-7)
Supplement: Supplementary file 4 — Reporting Summary [file 42003_2022_3521_MOESM4_ESM.pdf]

nature portfolio

Corresponding author(s): Alexandra Zhernakova

Last updated by author(s): Feb 14, 2022

Reporting Summary

Nature Portfolio wishes to improve the reproducibility of the work that we publish. This form provides structure for consistency and transparency in reporting. For further information on Nature Portfolio policies, see our Editorial Policies and the Editorial Policy Checklist.

Statistics

For all statistical analyses, confirm that the following items are present in the figure legend, table legend, main text, or Methods section.

n/a	Confirmed	
☐	☒	The exact sample size (*n*) for each experimental group/condition, given as a discrete number and unit of measurement
☐	☒	A statement on whether measurements were taken from distinct samples or whether the same sample was measured repeatedly
☐	☒	The statistical test(s) used AND whether they are one- or two-sided<br>*Only common tests should be described solely by name; describe more complex techniques in the Methods section.*
☐	☒	A description of all covariates tested
☐	☒	A description of any assumptions or corrections, such as tests of normality and adjustment for multiple comparisons
☐	☒	A full description of the statistical parameters including central tendency (e.g. means) or other basic estimates (e.g. regression coefficient) AND variation (e.g. standard deviation) or associated estimates of uncertainty (e.g. confidence intervals)
☐	☒	For null hypothesis testing, the test statistic (e.g. *F*, *t*, *r*) with confidence intervals, effect sizes, degrees of freedom and *P* value noted<br>*Give P values as exact values whenever suitable.*
☒	☐	For Bayesian analysis, information on the choice of priors and Markov chain Monte Carlo settings
☒	☐	For hierarchical and complex designs, identification of the appropriate level for tests and full reporting of outcomes
☐	☒	Estimates of effect sizes (e.g. Cohen's *d*, Pearson's *r*), indicating how they were calculated

Our web collection on statistics for biologists contains articles on many of the points above.

Software and code

Policy information about availability of computer code

Data collection	*Provide a description of all commercial, open source and custom code used to collect the data in this study, specifying the version used OR state that no software was used.*
Data analysis	Analysis scripts are publicly available at: https://github.com/GRONINGEN-MICROBIOME-CENTRE/Groningen768 Microbiome/tree/master/Projects/Telomere_analysis

For manuscripts utilizing custom algorithms or software that are central to the research but not yet described in published literature, software must be made available to editors and reviewers. We strongly encourage code deposition in a community repository (e.g. GitHub). See the Nature Portfolio guidelines for submitting code & software for further information.

Data

Policy information about availability of data

All manuscripts must include a data availability statement. This statement should provide the following information, where applicable:

- Accession codes, unique identifiers, or web links for publicly available datasets
- A description of any restrictions on data availability
- For clinical datasets or third party data, please ensure that the statement adheres to our policy

The data here presented belongs to Lifelines. Lifelines is specifically organized to make assessment results available for (re)use by third parties. The biological aging data used here, including sjTRECs expression, methylation age predictions, telomere length measurements, genetics, methylation and phenotypic data can be requested through Lifelines. A research proposal must be submitted for evaluation by the Lifelines Research Office.
Phenotypic data: Researchers must submit a data order (i.e. a selection of variables) and research proposal in the Lifelines online catalogue.
Omics data: Omics data are stored in the UMCG HPC. Omics data cannot be ordered using the Lifelines online catalogue, but are made accessible in full to

researchers with an approved request. With uniquely made linkage files the researcher can link an order of phenotypic data to omics data, if requested.
In addition to this, processed (de-anonymized) scRNA-seq data, including a text file that links each cell barcode to its respective individual, is available at the European Genome-Phenome Archive (EGA), under accession number EGAS EGAS00001005376.
GWAS summary statistics generated in this study are available at GWAS catalog, under the accession numbers: granulocytes_telomeres - GCST90101887 ; lymphocytes_telomeres - GCST90101888; naive_Tcells_telomeres - GCST90101889 ; memory_Tcells_telomeres - GCST90101890; Bcells_telomeres - GCST90101891 ; NKcells_telomeres - GCST90101892

Field-specific reporting

Please select the one below that is the best fit for your research. If you are not sure, read the appropriate sections before making your selection.

☒ Life sciences ☐ Behavioural & social sciences ☐ Ecological, evolutionary & environmental sciences

For a reference copy of the document with all sections, see nature.com/documents/nr-reporting-summary-flat.pdf

Life sciences study design

All studies must disclose on these points even when the disclosure is negative.

Sample size	No power analysis was performed. Samples available from the Lifelines Deep cohort were used for data generation and correlation.
Data exclusions	Samples in which telomere length measurement failed in at least one sample were removed.
Replication	No replication was performed due to the unique nature of the data used in the study.
Randomization	No analysis included a group comparison.
Blinding	No analysis included a group comparison.

Reporting for specific materials, systems and methods

We require information from authors about some types of materials, experimental systems and methods used in many studies. Here, indicate whether each material, system or method listed is relevant to your study. If you are not sure if a list item applies to your research, read the appropriate section before selecting a response.

Materials & experimental systems

n/a	Involved in the study	
☒	☐	Antibodies
☒	☐	Eukaryotic cell lines
☒	☐	Palaeontology and archaeology
☒	☐	Animals and other organisms
☐	☒	Human research participants
☒	☐	Clinical data
☒	☐	Dual use research of concern

Methods

n/a	Involved in the study	
☒	☐	ChIP-seq
☒	☐	Flow cytometry
☒	☐	MRI-based neuroimaging

Human research participants

Policy information about studies involving human research participants

Population characteristics	Lifelines is a multi-disciplinary prospective population-based cohort study examining, in a unique three-generation design, the health and health-related behaviours of 167,729 persons living in the North of the Netherlands. It employs a broad range of investigative procedures to assess the biomedical, socio-demographic, behavioural, physical and psychological factors that contribute to the health and disease of the general population, with a special focus on multi-morbidity and complex genetics41 506 . We collected data from the subcohort Lifelines DEEP (LLD, n =1,057, 57.6% female, mean age (including months) 43.9 years [range 18–81.4 years]). Extensive information on demographics, health and lifestyle factors including smoking and diet was collected via detailed questionnaires. Mean BMI of participants was 25.1 [range 15.8–44.9]. Common age-related diseases within the cohort included hypertension (23% of 841 participants with information), type 2 diabetes (1.3% of 1,039 participants with information) and hypercholesterolemia (14% of 900 participants with information). In the cohort, 20% of individuals smoked currently, 48% smoked for at least 1 year and 37% had mothers and 65% had fathers who smoked.
Recruitment	Samples were recruited as part of the Lifelines Deep cohort.
Ethics oversight	The Lifelines study was approved by the medical ethical committee from the University

Ethics oversight	Medical Center Groningen (METc number: 2017/152).

Note that full information on the approval of the study protocol must also be provided in the manuscript.
